# Genomic Prediction Based on SNP Functional Annotation Using Imputed Whole-Genome Sequence Data in Korean Hanwoo Cattle

**DOI:** 10.3389/fgene.2020.603822

**Published:** 2021-01-21

**Authors:** Bryan Irvine M. Lopez, Narae An, Krishnamoorthy Srikanth, Seunghwan Lee, Jae-Don Oh, Dong-Hyun Shin, Woncheoul Park, Han-Ha Chai, Jong-Eun Park, Dajeong Lim

**Affiliations:** ^1^Division of Animal Genomics and Bioinformatics, National Institute of Animal Science, Rural Development Administration, Wanju, South Korea; ^2^Department of Animal Science and Biotechnology, Chungnam National University, Daejeon, South Korea; ^3^Department of Animal Biotechnology, Chonbuk National University, Jeonju, South Korea; ^4^Department of Agricultural Convergence Technology, Chonbuk National University, Jeonju, South Korea

**Keywords:** genomic selection, pre-selected sequence variants, genome annotation, carcass traits, Hanwoo cattle

## Abstract

Whole-genome sequence (WGS) data are increasingly being applied into genomic predictions, offering a higher predictive ability by including causal mutations or single-nucleotide polymorphisms (SNPs) putatively in strong linkage disequilibrium with causal mutations affecting the trait. This study aimed to improve the predictive performance of the customized Hanwoo 50 k SNP panel for four carcass traits in commercial Hanwoo population by adding highly predictive variants from sequence data. A total of 16,892 Hanwoo cattle with phenotypes (i.e., backfat thickness, carcass weight, longissimus muscle area, and marbling score), 50 k genotypes, and WGS imputed genotypes were used. We partitioned imputed WGS data according to functional annotation [intergenic (IGR), intron (ITR), regulatory (REG), synonymous (SYN), and non-synonymous (NSY)] to characterize the genomic regions that will deliver higher predictive power for the traits investigated. Animals were assigned into two groups, the discovery set (7324 animals) used for predictive variant detection and the cross-validation set for genomic prediction. Genome-wide association studies were performed by trait to every genomic region and entire WGS data for the pre-selection of variants. Each set of pre-selected SNPs with different density (1000, 3000, 5000, or 10,000) were added to the 50 k genotypes separately and the predictive performance of each set of genotypes was assessed using the genomic best linear unbiased prediction (GBLUP). Results showed that the predictive performance of the customized Hanwoo 50 k SNP panel can be improved by the addition of pre-selected variants from the WGS data, particularly 3000 variants from each trait, which is then sufficient to improve the prediction accuracy for all traits. When 12,000 pre-selected variants (3000 variants from each trait) were added to the 50 k genotypes, the prediction accuracies increased by 9.9, 9.2, 6.4, and 4.7% for backfat thickness, carcass weight, longissimus muscle area, and marbling score compared to the regular 50 k SNP panel, respectively. In terms of prediction bias, regression coefficients for all sets of genotypes in all traits were close to 1, indicating an unbiased prediction. The strategy used to select variants based on functional annotation did not show a clear advantage compared to using whole-genome. Nonetheless, such pre-selected SNPs from the IGR region gave the highest improvement in prediction accuracy among genomic regions and the values were close to those obtained using the WGS data for all traits. We concluded that additional gain in prediction accuracy when using pre-selected variants appears to be trait-dependent, and using WGS data remained more accurate compared to using a specific genomic region.

## Introduction

The use of whole-genome sequence (WGS) data in genomic prediction is expected to be advantageous, since all or most of the causal mutations or single-nucleotide polymorphisms (SNPs) are putatively in strong linkage disequilibrium (LD) with causal mutations affecting the traits. This was confirmed in a simulation study (Meuwissen and Goddard, 2010), but in real data, the use of entirely WGS data was shown to lead to no or only small improvements in prediction accuracy. For instance, Heidaritabar et al. (2016) reported in chicken that increasing the marker density from 60 K SNP panel to imputed WGS data resulted in only a slight improvement (∼1%) in prediction accuracy. Frischknecht et al. (2018) also concluded that the inclusion of imputed WGS data did not lead to increase the accuracy of genomic prediction in Brown Swiss cattle.

The poor performance of using WGS data could be due to several reasons, including the small number of sequenced animals (Heidaritabar et al., 2016), imputation accuracy of marker genotypes (Druet et al., 2014), and LD between quantitative traits loci (QTL) and SNPs (Meuwissen and Goddard, 2010). Moreover, van den Berg et al. (2016) pointed out that such small improvement from using WGS data could be because only variants close to causative mutations or causative mutations themselves can improve genomic prediction accuracy. Thus, as an alternative to a simple increase in marker density, some studies suggested that the prediction accuracy could be improved by adding significant QTL or variants that were selected based on genome-wide association studies (GWAS) using WGS data (Brondum et al., 2015; Veerkamp et al., 2016; Moghaddar et al., 2019).

In recent years, the customized Hanwoo 50 k SNP Chip (58,990) has been the main technology used to calculate genomic breeding values (GEBV) in Hanwoo cattle. However, our previous study (Lopez et al., 2019) showed that only around 37,000 SNPs remained after genomic quality control for estimating the GEBV. SNPs were excluded mainly due to low minor allele frequency (MAF). Hence, these SNPs need to be replaced with those SNPs with high MAF likely to have an effect on breeding goal trait to further improve the accuracy of genomic prediction. Hayes et al. (2014) reported that variants in regulatory (REG) or coding regions could also have an effect on traits, since these variants (missense) have direct effects on proteins and are likely to have a phenotypic effect (Koufariotis et al., 2014). Our previous works also showed that the SNPs in REG regions were able to capture a large proportion of the total genetic variation in carcass traits of Hanwoo cattle (Bhuiyan et al., 2018; Srikanth et al., 2020). Therefore, incorporating such coding SNPs into genomic predictions may improve the prediction accuracy.

In this study, imputed WGS data were partitioned into different genomic regions based on the functional annotation information, namely, intergenic (IGR), intron (ITR), REG, synonymous (SYN), and non-synonymous (NSY). Then, GWAS was carried out for each genomic region and the entire WGS data for the pre-selection of variants in a separate discovery dataset. Each set of pre-selected variants were added to the standard 50 k SNP array separately, and the predictive performance of each of these SNP sets was evaluated. We investigated whether the use of variants selected based on their functional annotation information especially those from the coding regions would improve the prediction accuracy compared to those that were selected from the WGS data. Moreover, we evaluated the different density of pre-selected variants to identify the optimum number of variants to be added to the standard 50 k set. The ultimate goal of this study was to improve the predictive performance of the customized Hanwoo 50 k SNP panel by incorporating highly predictive variants from the imputed sequence data for backfat thickness, carcass weight, longissimus muscle area, and marbling score carcass traits from a commercial Hanwoo population.

## Materials and Methods

### Animals and Experimental Design

Data used in this study consisted of phenotypic and genotypic records of 16,892 Hanwoo cattle mostly composed of steers (16,535). These animals were born between April 2006 and June 2017 from around 4,000 farms in South Korea. Four routinely collected carcass traits were analyzed: backfat thickness (BFT in mm), carcass weight (CWT in kg), longissimus muscle area (LMA in cm^2^), and marbling score (MS: scored from 1 to 9). All animals were slaughtered at an average age of 30 months, and their carcass traits were measured in accordance with the guidelines proposed by the Korea Institute for Animal Production Quality Evaluation (KAPE). Ethics approval for this study was given by the Animal Care and Use Committee of the National Institute of Animal Science, Rural Development Administration, South Korea (2018–293). A detailed description of the data and other pertinent information were provided in the previous study of Lopez et al. (2019). The descriptive statistics for traits studied are shown in Table 1.

**TABLE 1:** Descriptive statistics for carcass traits in Hanwoo cattle.

**Trait^1^**	***N***	**Min**	**Max**	**Mean**	**SD**
BFT, mm	16,892	2.00	47.00	14.25	5.03
CWT, kg	16,892	159.00	692.00	441.06	52.31
LMA, cm^2^	16,892	34.00	156.00	95.61	12.06
MS (1–9)	16,892	1.00	9.00	6.10	1.87

*^1^Backfat thickness (BFT), carcass weight (CWT), longissimus muscle area (LMA), and marbling score (MS).*

The animals were divided into two non-overlapping groups, the discovery set used for variant detection in the GWAS and the cross-validation set for genomic prediction. To minimize any probable bias in the evaluation of prediction accuracy, the animals in the discovery set were composed of diverse individuals with low genetic relationships with those animals in the cross-validation set (Veerkamp et al., 2016). This was made possible by calculating the genomic relationship matrix (GRM) between all pairs of individuals in the population using GCTA (Yang et al., 2011), and then, a GRM threshold was applied to extract individuals from the dataset who do not have any relatives in population given the relatedness threshold. A different GRM threshold was tested as shown in Supplementary Table 1. A GRM threshold of 0.30 was used in this study to keep a reasonable number of animals in the two datasets, which was a little higher to the GRM threshold of 0.25 used by Moghaddar et al. (2019) for meat traits in Australian sheep populations. The GRM was constructed from 50 k SNP genotypes (SNPs were removed if they had a call rate lower than 0.90, MAF lower than 0.01, and Hardy–Weinberg disequilibrium < 0.000001). Ultimately, a total of 7,324 animals were assigned in the discovery set for GWAS while the remaining animals (9,568) were used in cross-validation for genomic prediction.

### Derivation of Corrected Phenotypes

Corrected phenotypes were used as response variables in genomic prediction analyses. Phenotypes were pre-corrected for fixed effects in a single-trait analysis using a pedigree-based model in the PREDICTF90 (Misztal et al., 2014):

y=Xb+Za+e,

where, **y** is a vector of observations; **b** is the vector of fixed effects of year-month of birth, slaughter year-month, slaughter place, herd (province-city/town), sex, and slaughter age as a covariate; **a** is the vector of random additive genetic effects $\left[a\,∼N\,\left(0,\,A\,\sigma_{\text{a}}^{2}\right)\right]$ where $\sigma_{\text{a}}^{2}$ is the additive genetic variance and **A** is the pedigree-based relationship matrix of individuals; **e** is the vector of random residual effect $\left[e\,∼N\,\left(0,\,I\,\sigma_{\text{e}}^{2}\right)\right]$ where $\sigma_{\text{e}}^{2}$ is the residual variance and **I** is an identity matrix. **X** and **Z** are indices matrices associating **b** and **a** to **y**.

### SNP Genotyping, Imputation, and Annotation

The genomic DNA for each animal was extracted from tissue samples using the DNeasy Blood and Tissue Kit (Qiagen, Valencia, CA, United States). Samples were genotyped using the customized Hanwoo 50 k SNP Chip (58,990) according to the manufacturer’s instructions (Illumina, South Korea). The following threshold levels were applied for quality control using the PLINK software (Purcell et al., 2007): SNPs were removed if they had a MAF < 0.01 or call rate <0.90 and Hardy–Weinberg disequilibrium <0.000001. Genotypes situated on the sex chromosomes were also excluded. Furthermore, individuals with more than 10% missing genotypes were removed. After applying these quality control measures, 37,712 SNPs were remained.

A two-step imputation process of 50 k genotypes to WGS data was performed using Minimac3 (Das et al., 2016) after pre-phasing the genotypes with Eagle v2.4.1. (Loh et al., 2016). Specifically, this entailed the imputation from the 50 k SNP chip to high-density (HD) genotypes using a reference set of 1166 animals genotyped with Bovine HD BeadChip (777,962 SNPs), which was subsequently followed by the imputation from HD genotypes to sequence data (26,936,924 SNPs) using a reference of 311 sequenced progeny tested Hanwoo bulls (Bhuiyan et al., 2018). The imputation accuracy used in this study was derived from the *r*^2^ value from Minimac3, which was the estimated value of the squared correlation between true and imputed genotypes. Similar to our previous studies (Bhuiyan et al., 2018; Srikanth et al., 2020), SNPs with an imputation accuracy (*r*^2^) lower than 0.60 were removed for further analysis. After removing those SNPs, the overall mean imputation accuracy was 0.87.

The imputed whole-genome variants were annotated based on the bovine genome assembly UMD 3.1 (Elsik et al., 2016). SNPs were annotated, filtered, and partitioned using SnpEff version 4.3 (Cingolani et al., 2012) and SnpSift software (Ruden et al., 2012). SNPs were partitioned into five genomic regions: IGR, ITR, regulatory (REG), SYN, and NSY. In addition, the combination of regulatory, synonymous and non-synonymous (RSN) SNPs was also considered in this study. Synonymous and NSY SNPs are in protein-coding regions (i.e., exons of genes), in which synonymous SNPs are those coding SNPs that does not modify the resulting amino acid while NSY SNPs alter the encoded amino acid. Regulatory SNPs are protein coding SNPs within 5-kb upstream of a gene. Other details of the functional classifications can be found in our previous works (Bhuiyan et al., 2018; Srikanth et al., 2020). After partitioning, the same quality control criteria employed for the 50 k panel described above were applied to each genomic region and WGS data. In addition, random pair of SNPs that were in high LD (*r*^2^ > 0.95) in a 5000-kb sliding window with 100 variants were excluded using the same software. The number of variants annotated in different genomic regions before and after quality control and after LD pruning is shown in Table 2.

**TABLE 2:** Number of variants annotated in different genomic regions before and after quality control and after LD pruning.

**Genomic region^1^**	**Before quality control**	**After quality control**	**After LD pruning**
50 k	58,990	37,712	–
WGS	13,502,733	11,948,082	1,561,308
IGR	9,436,699	8,307,710	1,115,262
ITR	3,936,080	3,436,573	523,494
REG	968,519	852,187	197,513
SYN	59,569	52,828	32,884
NSY	27,187	23,046	15,986
RSN	1,030,239	918,307	216,944

*^1^Whole-genome sequence (WGS), intergenic (IGR), intron (ITR), regulatory (REG), synonymous (SYN), non-synonymous (NSY), and combination of REG, SYN and NSY (RSN).*

### Pre-selection of Sequence Variants

Variant selection was based on their *p*-value from GWAS conducted on the discovery set (7324 animals). GWAS was performed both in WGS and each genomic region separately (IGR, ITR, REG, SYN, NSY, and RSN). Thus, we ran a total of seven GWAS for each trait using the mixed linear model based association analysis (MLMA) in the package GCTA (Yang et al., 2011). The model was:

$${\text{y}}_{c}=1\,\mu +{\text{s}}_{i}\,\alpha_{i}+\text{Zg}+\text{e},$$

where **y_*c*_** is a vector of corrected phenotypes; **μ** is the overall mean; **1** is a vector of ones; **s_*i*_** is a vector of genotypes for SNP_*i*_ (coded as 0, 1, or 2); α_*i*_ is the size of the effect of the marker (allele substitution effect); **g** is a vector of the GEBV of all individuals $\left[g\,∼N\,\left(0,\,G\,\sigma_{\text{g}}^{2}\right)\right],$ where $\sigma_{\text{g}}^{2}$ is the additive genetic variance and **G** is the marker-based GRM (VanRaden, 2008) constructed from different sets of genotypes (i.e., WGS, IGR, ITR, REG, SYN, NSY and RSN); **Z** is an incidence matrix linking **g** to **y_*c*_**; and **e** is the vector of random residual effect $\left[e\,∼N\,\left(0,\,I\,\sigma_{\text{e}}^{2}\right)\right].$

We selected 1,000, 3,000, 5,000, and 10,000 SNPs with the lowest *p-*values from both WGS and each genomic region. In addition, we randomly select the same number of added SNPs from WGS (RAN). Each set of pre-selected SNPs were added to the 50 k panel separately and tested for genomic prediction. Pre-selected SNPs that overlapped with those on the 50 k SNP chip was retained in the 50 k set and replaced by others to keep the same number of added SNPs.

### Genomic Prediction

The GEBVs of all genotyped individuals in the cross-validation dataset were predicted with the genomic best linear unbiased prediction (GBLUP) model using MTG2 (Lee and Van der Werf, 2016). Genomic relationship matrices (GRM) were constructed for each of the genotype sets: 50 k, 50 k + WGS, 50 k + IGR, 50 k + ITR, 50 k + REG, 50 k + SYN, 50 k + NSY, 50 k + RSN, and 50 k + RAN. The added variants to the 50 k panel have different densities of 1,000, 3,000, 5,000, or 10,000. A single-trait animal model was fitted as follows:

$${\text{y}}_{c}=1\,\mu +\text{Zg}+\text{e},$$

where the terms are as defined in the GWAS model above. Variance components were estimated with restricted maximum likelihood algorithm as employed in MTG2.

A 10-fold cross-validation scheme was utilized to determine prediction accuracy. The cross-validation dataset (9,568 animals) was randomly partitioned into 10 groups without overlapping of samples. In each cross-validation, one group (∼956) was treated as the validation set and the remaining groups were used as the training set (∼8,612). Phenotypes of animals in the validation set were assumed as unknown. The accuracy of genomic prediction was calculated as the correlation between the GEBV of the validation set and their corrected phenotypes, divided by the square root of heritability of the trait (estimated using a 50 K SNP data). Furthermore, the unbiasedness of genomic prediction was evaluated using the regression coefficient of corrected phenotypes on GEBV.

## Results

### Heritability Estimates

Heritability estimates for carcass traits were obtained by fitting the different GRM constructed from various SNP sets as shown in Table 3. These estimates for the four carcass traits investigated were medium to high ranging from 0.32 to 0.40 based on the GRM from the regular 50 k panel. After the inclusion of pre-selected SNPs from different sets of markers, heritability estimates were generally similar for all traits compared to the 50 k genotypes. However, while we observed that the heritability estimates for BFT, LMA, and MS showed no notable change when more pre-selected SNPs were added from the WGS, a small decrease was noted for CWT. Meanwhile, the addition of 10,000 pre-selected SNPs to the 50 k set slightly increase the heritability for MS especially those from REG, SYN, or RSN.

**TABLE 3:** Estimates of heritability for carcass traits of Hanwoo cattle based on different SNP sets.*

**Trait^1^**	**Number of added variants**	**50 k**	**50 k+**							
			**WGS**	**IGR**	**ITR**	**REG**	**SYN**	**NSY**	**RSN**	**RAN**
BFT	1000	0.32	0.33	0.33	0.33	0.33	0.33	0.32	0.33	0.32
	3000		0.33	0.33	0.33	0.33	0.33	0.33	0.33	0.32
	5000		0.33	0.33	0.33	0.33	0.33	0.33	0.33	0.33
	10,000		0.33	0.33	0.33	0.33	0.34	0.33	0.33	0.33
CWT	1000	0.33	0.32	0.32	0.33	0.33	0.34	0.34	0.33	0.33
	3000		0.31	0.31	0.33	0.33	0.34	0.34	0.34	0.34
	5000		0.31	0.31	0.32	0.33	0.34	0.34	0.34	0.34
	10,000		0.30	0.31	0.33	0.34	0.34	0.34	0.34	0.34
LMA	1000	0.32	0.32	0.32	0.32	0.32	0.32	0.32	0.32	0.32
	3000		0.32	0.32	0.32	0.33	0.32	0.32	0.33	0.32
	5000		0.32	0.32	0.32	0.33	0.33	0.33	0.33	0.32
	10,000		0.32	0.32	0.32	0.33	0.33	0.33	0.33	0.32
MS	1000	0.40	0.41	0.41	0.41	0.42	0.41	0.41	0.42	0.41
	3000		0.42	0.42	0.42	0.42	0.42	0.42	0.42	0.41
	5000		0.42	0.42	0.42	0.42	0.42	0.42	0.42	0.41
	10,000		0.42	0.42	0.42	0.43	0.43	0.42	0.43	0.42

Whole-genome sequence (WGS), intergenic (IGR), intron (ITR), regulatory (REG), synonymous (SYN), non-synonymous (NSY), combination of regulatory, synonymous, non-synonymous (RSN) and randomly selected from WGS (RAN).^1^Backfat thickness (BFT), carcass weight (CWT), longissimus muscle area (LMA), and marbling score (MS).* Standard error of heritability was between 0.015 and 0.018.

### Genomic Prediction Using Trait-Specific Pre-selected Variants

**FIGURE 1:**
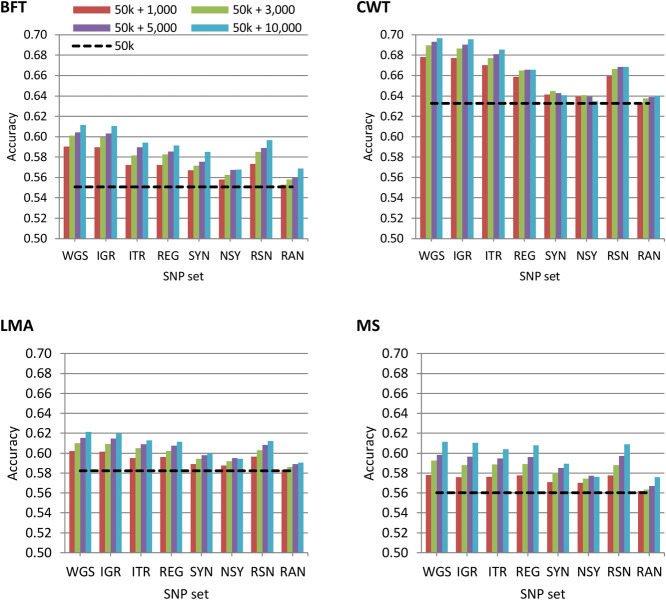
Accuracy of genomic predictions using the 50 k SNP panel with trait-specific pre-selected variants from different genomic regions. The added variants to the 50 k panel have different densities of 1,000, 3,000, 5,000, or 10,000. The black dashed line indicates the accuracy of prediction using the 50 k SNP panel only. The traits were backfat thickness (BFT), carcass weight (CWT), longissimus muscle area (LMA), and marbling score (MS). Whole-genome sequence (WGS); intergenic (IGR); intron (ITR); regulatory (REG); synonymous (SYN); non-synonymous (NSY); combination of regulatory, synonymous, non-synonymous (RSN); and randomly selected from WGS (RAN).

The accuracy of genomic prediction using the regular 50 k SNP panel and 50 k with trait-specific pre-selected variants from WGS, IGR, ITR, REG, SYN, NSY, RSN, or RAN is shown in Figure 1. In general, the results showed that the predictive performance of the regular 50 k SNP panel can be improved by adding pre-selected variants from imputed sequence data. However, this improvement in prediction accuracy varied among traits. When using GRM constructed on a 50 k panel, genomic prediction accuracies for BFT, CWT, LMA, and MS were 0.55, 0.63, 0.58, and 0.56, respectively. These values further increased to 7.1 to 11%, 7.2 to 10%, 3.4 to 6.7%, and 3.2 to 9.1% for BFT, CWT, LMA, and MS, correspondingly when 1,000 to 10,000 pre-selected SNPs from WGS were added to the 50 k set. However, it should be noted that there was only a slight improvement in prediction accuracy when adding either 3000 or 5000 variants for most traits, though in most cases, the addition of a higher number of pre-selected variants to 50 k tended to increase the prediction accuracy for all traits.

Using pre-selected variants from a specific genomic region based on their functional annotation did not show a clear advantage compared to using the whole genome. Hence, prediction accuracy based on 50 k + WGS remained the most accurate among SNP sets considered in this study. Nonetheless, prediction accuracies achieved by each of the genomic regions were substantially higher than those of randomly pre-selected SNPs from WGS for all traits. Moreover, we observed that the impact of genomic regions on the prediction accuracy differed between traits. The prediction accuracies based on 50 k + IGR were the most similar to those of 50 k + WGS for BFT, CWT, and LMA while 50 k + IGR, 50 k + ITR, 50 k + REG, or 50 k + RSN for MS. Meanwhile, 50 k plus the pre-selected variants from SYN or NSY consistently yielded the lowest prediction accuracy among the genomic regions.

**FIGURE 2:**
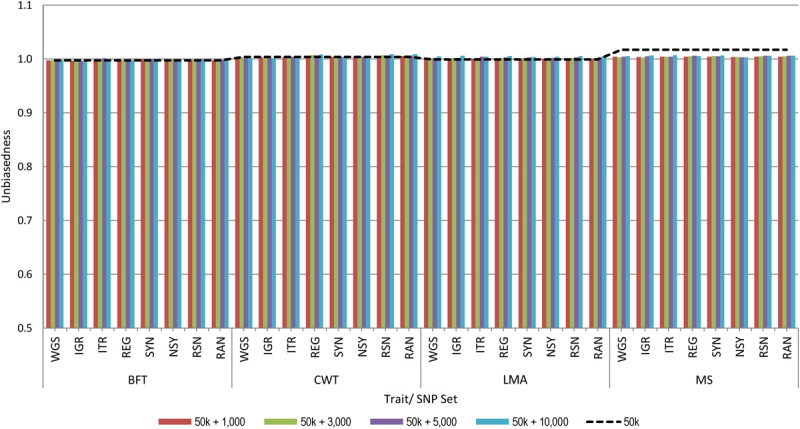
Unbiasedness of genomic predictions using 50 k SNP panel with trait-specific pre-selected variants from different genomic regions. The added variants to the 50 k panel have different densities of 1,000, 3,000, 5,000, or 10,000. The black dashed line indicates the unbiasedness of prediction using the 50 k SNP panel only. These traits were backfat thickness (BFT), carcass weight (CWT), longissimus muscle area (LMA), and marbling score (MS). Whole-genome sequence (WGS); intergenic (IGR); intron (ITR); regulatory (REG); synonymous (SYN); non-synonymous (NSY); combination of regulatory, synonymous, non-synonymous (RSN); and randomly selected from WGS (RAN).

Figure 2 shows the unbiasedness of genomic prediction assessed as the regression coefficient of the corrected phenotypes on GEBV in the validation population. The regression coefficients for all traits based on the 50 k SNP panel were very close to 1, ranging from 1.00 to 1.02, which indicates unbiased prediction. When pre-selected variants were added to the 50 k from any set of genotypes considered in this study, regression coefficients were similar with those of the 50 k set for all traits. Moreover, we observed that increasing the number of added variants to the 50 k genotypes did not have a considerable effect on the prediction bias estimates. These results then suggest that the estimates of prediction bias were not affected by the strategy used for the pre-selection of sequence variants.

### Genomic Prediction Using Combined Pre-selected Variants

**FIGURE 3:**
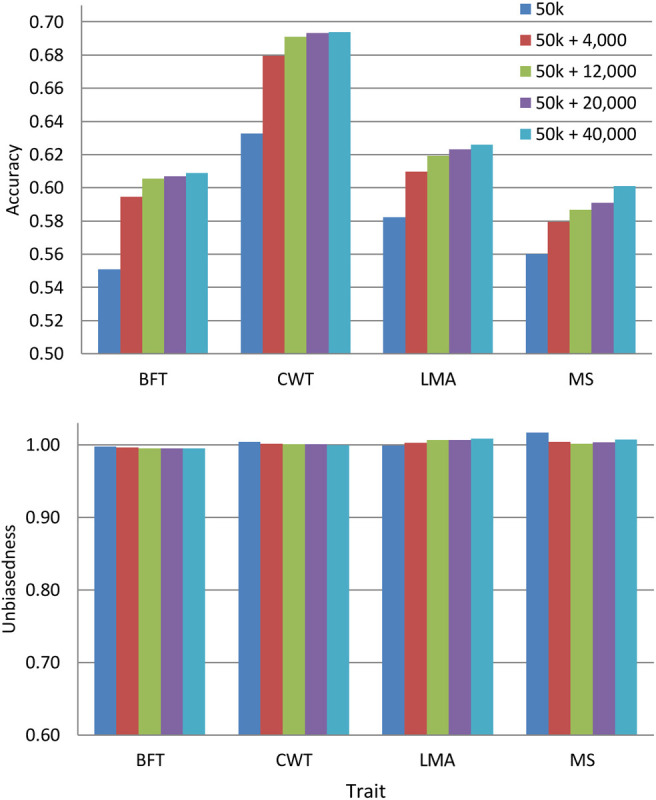
Accuracy and unbiasedness of genomic predictions using the regular 50 k SNP panel and 50 k with the combined pre-selected variants from whole-genome sequence (1,000, 3,000, 5,000, or 10,000 variants from each trait). These variants were pre-selected from entire whole-genome sequence data. These traits were backfat thickness (BFT), carcass weight (CWT), longissimus muscle area (LMA), and marbling score (MS).

Since the pre-selected variants differed among traits, we evaluated the predictive performance by combining such variants from all traits with 50 k genotypes. We only used the pre-selected variants from WGS since they improve the prediction accuracy more than those from a specific genomic region. The overlapping of pre-selected variants between traits is provided in Supplementary Figure 1. Figure 3 shows the prediction accuracy and unbiasedness of using the combined pre-selected variants from all traits. After the inclusion of 4,000 pre-selected variants (1,000 variants from each trait) to the 50 k set, the average prediction accuracy across traits improved by 5.9%. The prediction accuracy was further increased by including more variants, but the additional gain was only marginal after the addition of 12,000 more variants for most traits. Specifically, the average across traits improvement was 7.5, 8.1, and 8.7% when 12,000, 20,000, and 40,000 combined pre-selected variants were added to the 50 k genotypes, respectively. In terms of prediction bias, the results were similar to those presented above for 50 k + WGS, where regression coefficients for all traits were very close to 1.

## Discussion

In this study, we investigated the use of variants that were selected from imputed WGS data with the hope of improving the predictive performance of regular 50 k SNP panel for carcass traits in commercial Hanwoo population. We also evaluated the variants pre-selected based on their functional annotation information. The heritability estimates, genomic prediction accuracy, and unbiasedness based on 50 k plus pre-selected variants from different genomic regions (WGS, IGR, ITR, REG, SYN, NSY, or RSN) were compared to those of the standard 50 k genotypes for all traits.

Heritability estimates based on standard 50 k genotypes for BFT (0.32), LMA (0.32), and MS (0.40) in this study were in agreement with those observed by Lee et al. (2018) for a population of 119,545 Hanwoo cattle. On the other hand, the estimated heritability for CWT was 0.33 in this study and 0.42 in the work of Lee et al. (2018). High heritability estimates (except CWT = 0.31) were also observed by Mehrban et al. (2019) for BFT (0.50), LMA (0.44), and MS (0.61) using 5,824 Hanwoo steers. The observed differences between the heritability estimates in this present and other previous studies might be attributed to factors such as different number of animals and fixed effects used.

The effect of adding pre-selected SNPs to the 50 k array on the heritability estimate varied among traits and SNP sets within traits. According to Jensen et al. (2012), the amount of additive genetic variance that genomic markers can explain depends on several aspects such as (1) number of markers on causative sites, (2) markers in LD with causative genes due to close “historical” linkage at the population level, and (3) LD among markers and genes at the family level, due to the family structure in the population. The slight increase in heritability estimates of MS after the addition of 10,000 pre-selected SNPs to the 50 k set could conceivably be due to the potential linkage of the additional markers to causal variants. This finding is comparable to a previous study in sheep (Moghaddar et al., 2019) that also reported a slight increase in heritability when pre-selected variants were added to the 50 k set. Meanwhile, Raymond et al. (2018b) reported no increase in heritability estimates after adding pre-selected SNPs to the 50 k set for stature in dairy cattle breeds. In case of CWT, the minimal decrease in heritability was likely due to those added SNPs (e.g., WGS) that were more concentrated in some regions as shown in Supplementary Figure 2B. This is because the estimation of heritability is primarily driven by (higher) relationships between animals, in which case markers not distributed across the whole genome could less precisely capture the family relationships between individuals. Contrastingly, added SNPs (e.g., SYN or NSY) that were less concentrated in some regions and distributed across the whole genome can contribute in estimating heritabilities more accurately. Raymond et al. (2018b) pointed out that the selected variants may explain only a small amount of the genetic variation due to their small effects, but rather can contribute considerably to genomic prediction.

The prediction accuracy for all carcass traits increases with the addition of pre-selected SNPs to the 50 k panel. Previous works have also described that integrating these pre-selected markers derived from sequence data into a medium (50 k or 80 k) density panel data can improve the accuracy of genomic prediction (Brondum et al., 2015; Al Kalaldeh et al., 2019; Liu et al., 2019). Moreover, the additional gain in prediction accuracy also varied among traits as shown in Figure 1. This is expected due to the differences in the genetic architecture of each trait, i.e., if a trait is affected by relatively few QTL (each with a relatively large effect), using pre-selected SNPs would be more advantageous. Our previous study (Lopez et al., 2019) showed that CWT is controlled by few QTLs with large effects, thus benefitting the most when pre-selected SNPs were used.

Compared to using pre-selected SNPs from specific genomic regions, utilizing WGS data resulted in higher prediction accuracy for all traits of Hanwoo cattle. In commercial chicken population, Morota et al. (2014) also demonstrated that the predictive performance of the WGS remained more accurate compared to that of specific genomic regions. However, they used a different number of markers when comparing those sets of genotypes that might have affected their predictive performance results. Contradictory to this, Xu et al. (2020) reported higher prediction accuracies using functional annotation information of the gene class (coding regions) with a haplotype-based model as compared to using all markers for three carcass traits in Chinese Simmental beef cattle. These discrepancies with our results can be due to the different methodologies employed across studies. In this study, we only used the GBLUP method because this is the routinely applied genetic evaluation procedure in national and commercial breeding programs of Hanwoo cattle. However, further work is recommended for the inclusion of functional annotation data into the genomic prediction using a more complex model such as the haplotype-based model.

**FIGURE 4:**
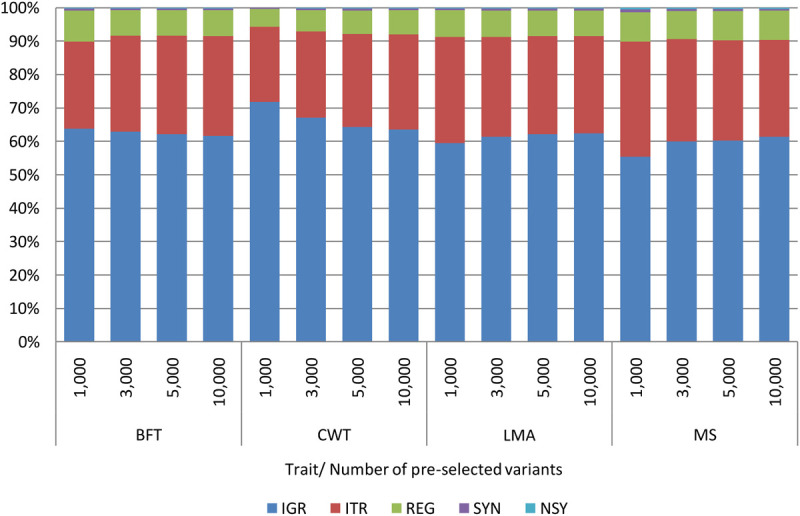
Functional classification of 1,000, 3,000, 5,000, and 10,000 pre-selected variants from WGS by trait. Backfat thickness (BFT), carcass weight (CWT), longissimus muscle area (LMA), and marbling score (MS). Intergenic (IGR), intron (ITR), regulatory (REG), synonymous (SYN), and non-synonymous (NSY).

Comparable with the findings of previous studies (Morota et al., 2014; Xu et al., 2020), some genome regions provided higher prediction accuracy than the others. Among genomic regions, pre-selected SNPs from the IGR gave the highest prediction accuracy for most of the traits. Besides, the prediction accuracies achieved by IGR were very close to those of WGS. Contrary to our expectations, selected SNPs from the coding regions (REG, SYN, or NSY) did not show better predictive performance over SNPs from other genomic regions. Heidaritabar et al. (2016) had also found lower prediction accuracy in commercial chicken population with coding SNPs SYN compared to the non-coding (IGR) when a similar number of SNPs were used for prediction. On the other hand, Morota et al. (2014) revealed that the predictive performance of coding SNPs for the ultrasound area of breast meat was better than that of IGR SNPs with IGR being better than the coding regions for body weight and hen house egg production traits. To further evaluate the contribution of variants from the IGR in this study, the functional classifications of pre-selected variants from WGS were determined. As shown in Figure 4, a high percentage of pre-selected SNPs were located in the IGR for all traits; specifically, around 60% of the 10,000 pre-selected SNPs from each trait were found in this region. With this, explorations should not be limited to variants within the coding regions alone as non-coding genomic regions are also equally important as they also affect some traits of interest (Abdollahi-Arpanahi et al., 2016).

In this study, prediction bias was assessed as regression coefficients of corrected phenotypes on GEBV. Results showed that all regression coefficients were around 1 for any set of genotypes for all traits, indicating that our predictions were unbiased. Moreover, these findings suggest that prediction bias was not affected by the strategy used for pre-selecting variants or the density of the added variants. Here, we used a separate group of animals for the discovery set having low genetic relationships with those in the cross-validation set. Previous work reported an increased prediction bias due to the overlaps between the validation and discovery datasets (Veerkamp et al., 2016).

In contrast to other studies (Raymond et al., 2018a; Moghaddar et al., 2019), we did not set any specific threshold for the *p*-value of marker effects for the selection of sequence variants because setting a specific *p*-value threshold resulted in unequal numbers of selected variants between genomic regions, and a previous study indicated that the density of the SNP panel influences the accuracy of genomic prediction (Daetwyler et al., 2010). Instead, we ranked the SNPs based on *p* value, selected the top 1,000, 3,000, 5,000, or 10,000 SNPs, and investigated the optimum number of selected variants to be used. In general, the prediction accuracy continuously increased as the added SNPs increased. However, we observed that the additional gain in prediction accuracy between 3,000 and 5,000 SNPs was very close for most of the traits. Also, the improvement in prediction accuracy between 3,000 and 10,000 SNPs was only 0.01 (2%) on the average. Thus, we conclude that adding at least 3,000 SNPs to the Hanwoo 50 k SNP panel from each trait is sufficient enough to improve the prediction accuracy. This was further confirmed after evaluating the prediction accuracy of the combined pre-selected variants from whole-genome data from all traits wherein the prediction accuracies upon the addition of ∼12,000 pre-selected variants (3,000 variants from each trait) were similar to those of 20,000 or 40,000 pre-selected variants for most of the traits (Figure 3). Previous studies have demonstrated that moderate marker density is adequate to accurately estimate the relationship between animals in the population compared to higher-density genotypes (Su et al., 2012; VanRaden et al., 2013; Heidaritabar et al., 2016).

In conclusion, our results indicate that the predictive performance of the customized Hanwoo 50 k SNP panel can be improved by integrating highly predictive sequence variants of 3000 SNPs from each trait from the WGS data. A substantial improvement in the prediction accuracy was observed for CWT and BFT while only a slight improvement was noted for LMA and MS, implying that the extra gain in prediction accuracy from the pre-selected variants appears to be trait-dependent. Moreover, the strategy in selecting variants based on their functional annotation did not show any clear advantage compared to using WGS data. However, further research is recommended for the inclusion of functional annotation data into the genomic prediction using a more complex model such as the haplotype-based model.

## Data Availability Statement

The datasets presented in this study can be found in online repositories. The names of the repository/repositories and accession number(s) can be found below: http://nabic.rda.go.kr/ostd/basic/snpVcfView.do?selectedId=NV-0618-000001.

## Ethics Statement

The animal study was reviewed and approved by the Institutional Animal Care and Use Committee, National Institute of Animal Science, Rural Development Administration, Republic of Korea.

## Author Contributions

DL conceived and supervised the study. DL, BL, and KS designed the study. SL, DH-S, and J-DO provided the data for analysis. KS and NA performed the imputation and annotation. WP, H-HC, and J-EP contributed to quality control of genotype data. BL and NA conducted the statistical analyses and drafted the manuscript. DL and KS edited and improved the manuscript. All authors read and approved the manuscript.

## Conflict of Interest

The authors declare that the research was conducted in the absence of any commercial or financial relationships that could be construed as a potential conflict of interest.

## Supplementary Material

Supplementary Figure 1 Venn diagram of 1,000 **(A)**, 3,000 **(B)**, 5,000 **(C)**, and 10,000 **(D)** pre-selected variants from WGS overlapping between traits. Backfat thickness (BFT), carcass weight (CWT), longissimus muscle area (LMA), and marbling score (MS).

Supplementary Figure 2A Manhattan plot of genome-wide association study (GWAS) using different set of SNPs for backfat thickness (BFT) pre-selected variants from entire whole-genome sequence data overlapping between traits. Whole-genome sequence (WGS), intergenic (IGR), intron (ITR), regulatory (REG), synonymous (SYN), non-synonymous (NSY), and combination of regulatory, synonymous and non-synonymous (RSN).

Supplementary Figure 2B Manhattan plot of genome-wide association study (GWAS) using different set of SNPs for carcass weight (CWT) pre-selected variants from entire whole-genome sequence data overlapping between traits. Whole-genome sequence (WGS), intergenic (IGR), intron (ITR), regulatory (REG), synonymous (SYN), non-synonymous (NSY), and combination of regulatory, synonymous and non-synonymous (RSN).

Supplementary Figure 2C Manhattan plot of genome-wide association study (GWAS) using different set of SNPs for longissimus muscle area (LMA) pre-selected variants from entire whole-genome sequence data overlapping between traits. Whole-genome sequence (WGS), intergenic (IGR), intron (ITR), regulatory (REG), synonymous (SYN), non-synonymous (NSY), and combination of regulatory, synonymous and non-synonymous (RSN).

Supplementary Figure 2D Manhattan plot of genome-wide association study (GWAS) using different set of SNPs for marbling score (MS) pre-selected variants from entire whole-genome sequence data overlapping between traits. Whole-genome sequence (WGS), intergenic (IGR), intron (ITR), regulatory (REG), synonymous (SYN), non-synonymous (NSY), and combination of regulatory, synonymous and non-synonymous (RSN).

Supplementary Table 1 Number of singleton animals based on different GRM relatedness threshold.
