# Supplementary figures and images for: Genomic Prediction Based on SNP Functional Annotation Using Imputed Whole-Genome Sequence Data in Korean Hanwoo Cattle
Source: Front Genet. 2021 Jan 21;11:603822. doi: 10.3389/fgene.2020.603822 (PMC7859490; doi:10.3389/fgene.2020.603822)

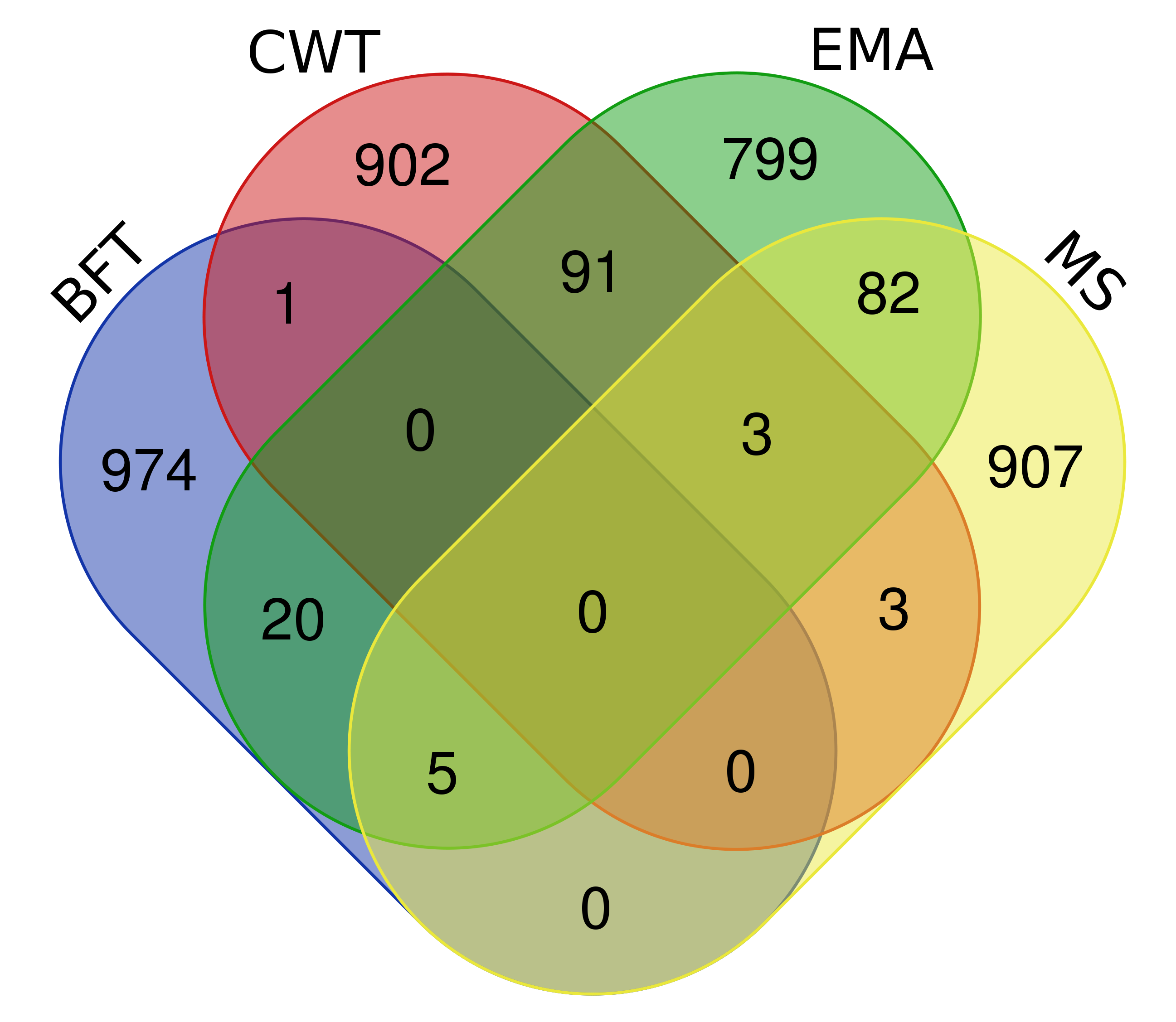

Supplement: Supplementary Figure 1 — Venn diagram of 1,000 (A), 3,000 (B), 5,000 (C), and 10,000 (D) pre-selected variants from WGS overlapping between traits. Backfat thickness (BFT), carcass weight (CWT), longissimus muscle area (LMA), and marbling score (MS). [file Data_Sheet_1.ZIP › Figure 1 - Venn_tif/Figure 1A.tiff]

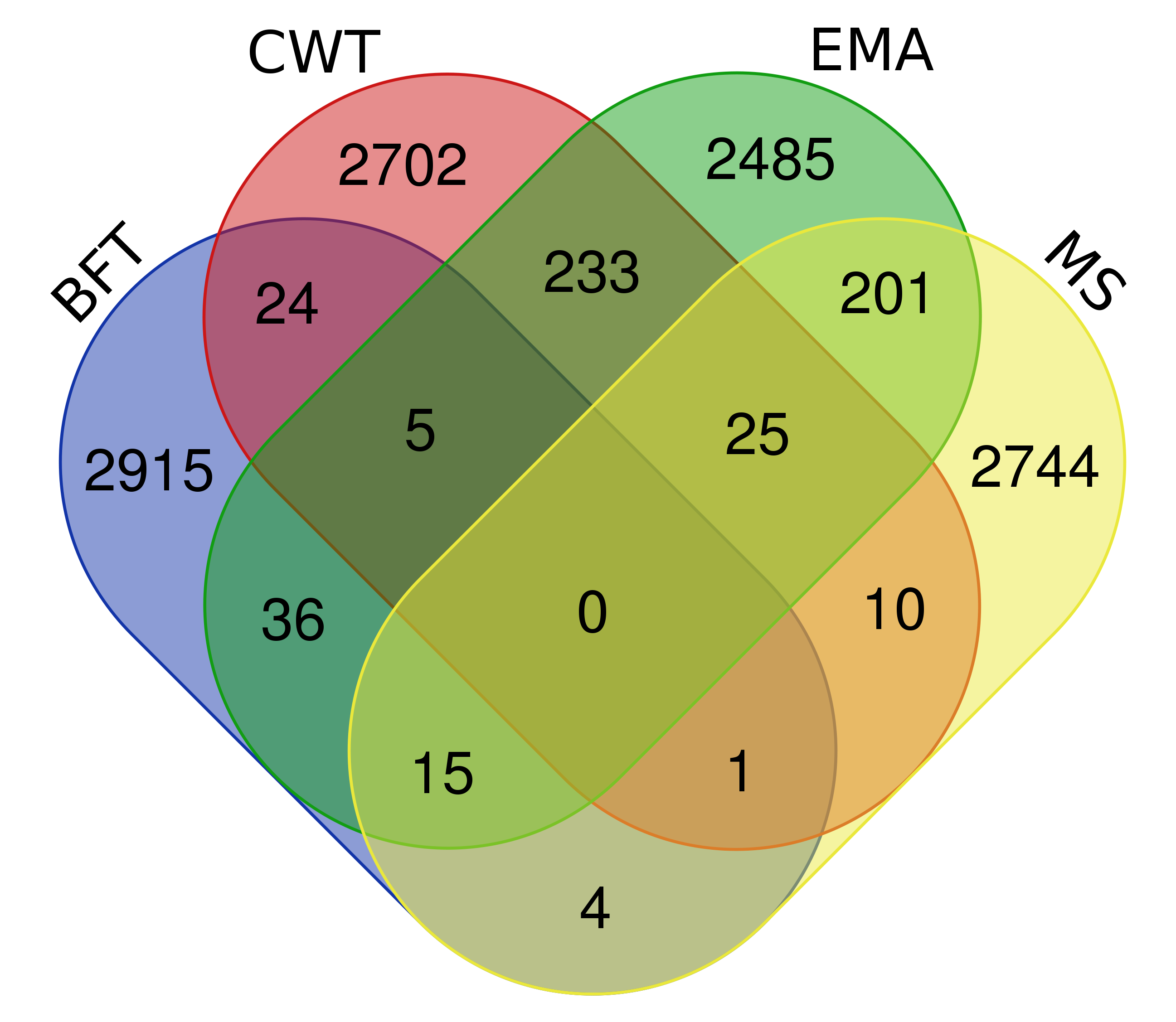

Supplement: Supplementary Figure 1 — Venn diagram of 1,000 (A), 3,000 (B), 5,000 (C), and 10,000 (D) pre-selected variants from WGS overlapping between traits. Backfat thickness (BFT), carcass weight (CWT), longissimus muscle area (LMA), and marbling score (MS). [file Data_Sheet_1.ZIP › Figure 1 - Venn_tif/Figure 1B.tiff]

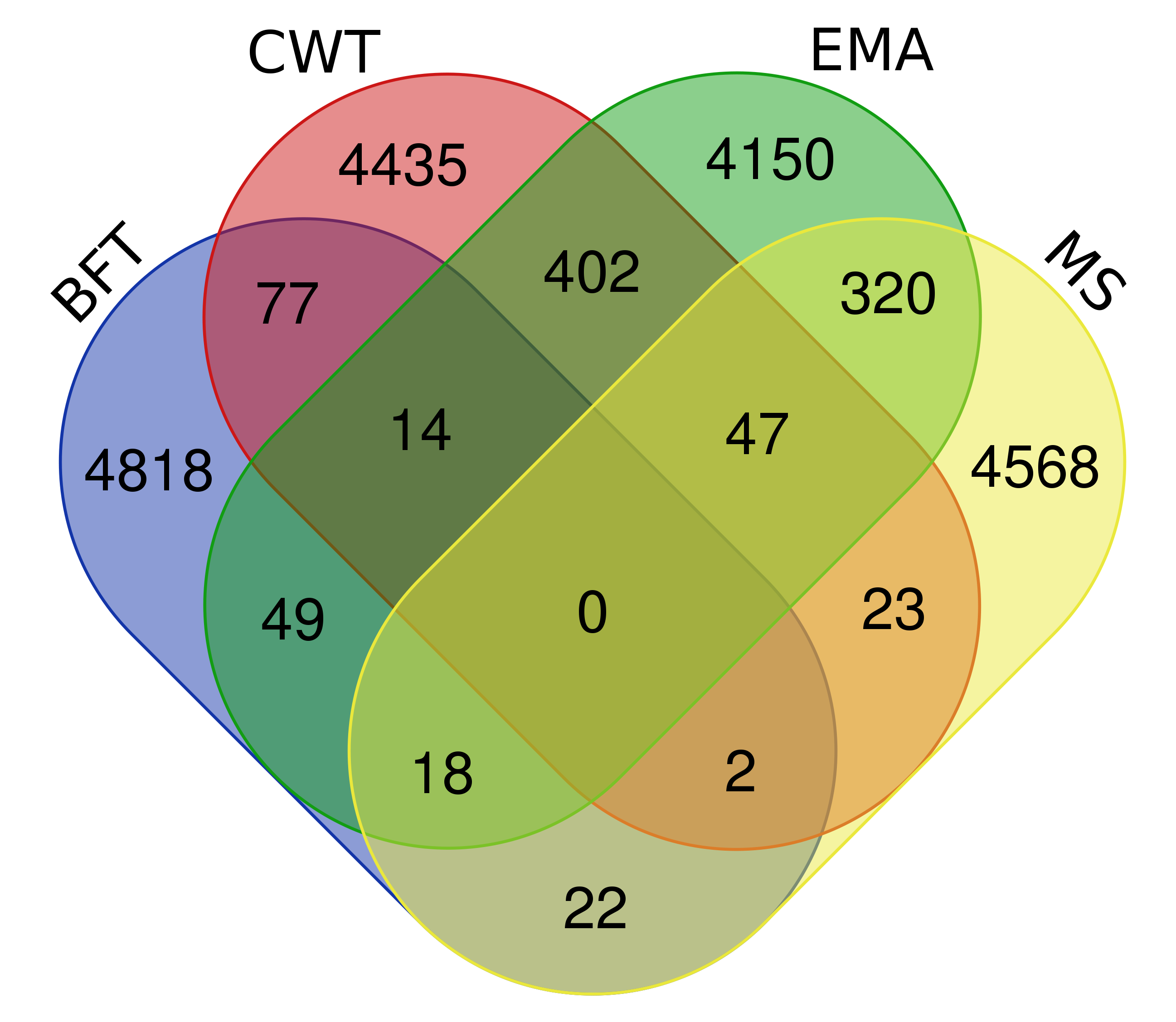

Supplement: Supplementary Figure 1 — Venn diagram of 1,000 (A), 3,000 (B), 5,000 (C), and 10,000 (D) pre-selected variants from WGS overlapping between traits. Backfat thickness (BFT), carcass weight (CWT), longissimus muscle area (LMA), and marbling score (MS). [file Data_Sheet_1.ZIP › Figure 1 - Venn_tif/Figure 1C.tiff]

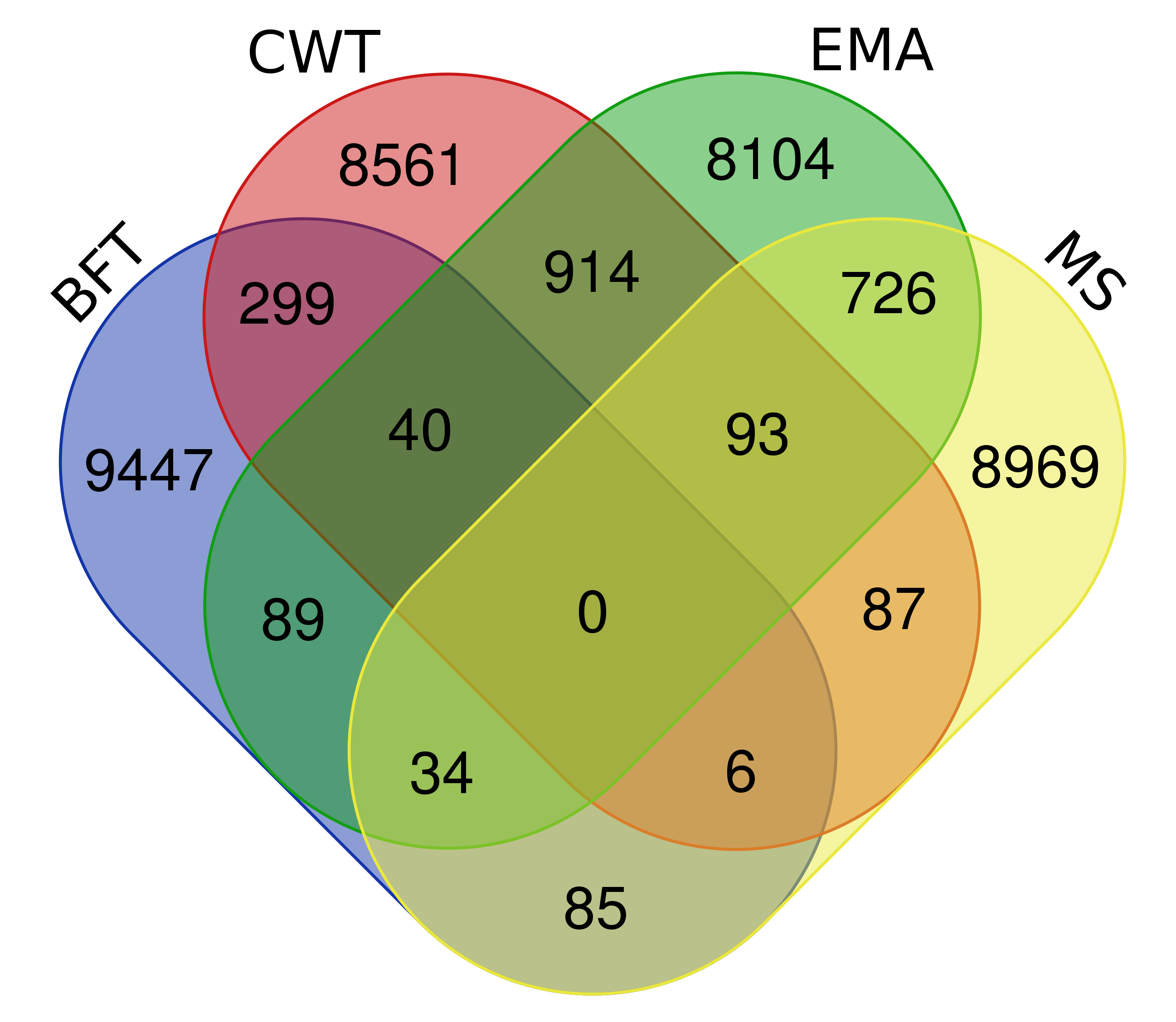

Supplement: Supplementary Figure 1 — Venn diagram of 1,000 (A), 3,000 (B), 5,000 (C), and 10,000 (D) pre-selected variants from WGS overlapping between traits. Backfat thickness (BFT), carcass weight (CWT), longissimus muscle area (LMA), and marbling score (MS). [file Data_Sheet_1.ZIP › Figure 1 - Venn_tif/Figure 1D.tiff]

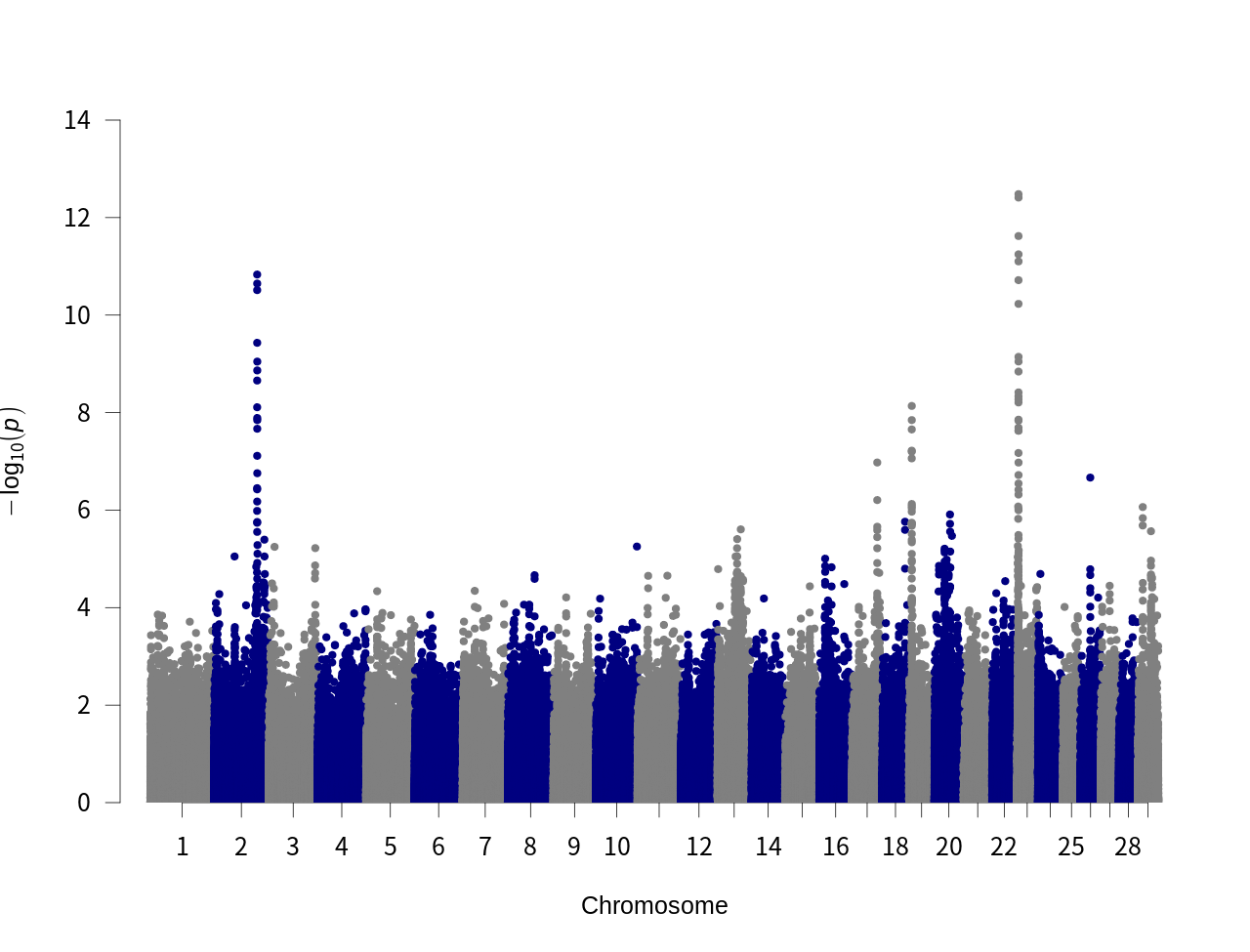

Supplement: Supplementary Figure 1 — Venn diagram of 1,000 (A), 3,000 (B), 5,000 (C), and 10,000 (D) pre-selected variants from WGS overlapping between traits. Backfat thickness (BFT), carcass weight (CWT), longissimus muscle area (LMA), and marbling score (MS). [file Data_Sheet_1.ZIP › Figure 2 - Manhattan_tif/Figure 2A - IGR_BFT.tiff]

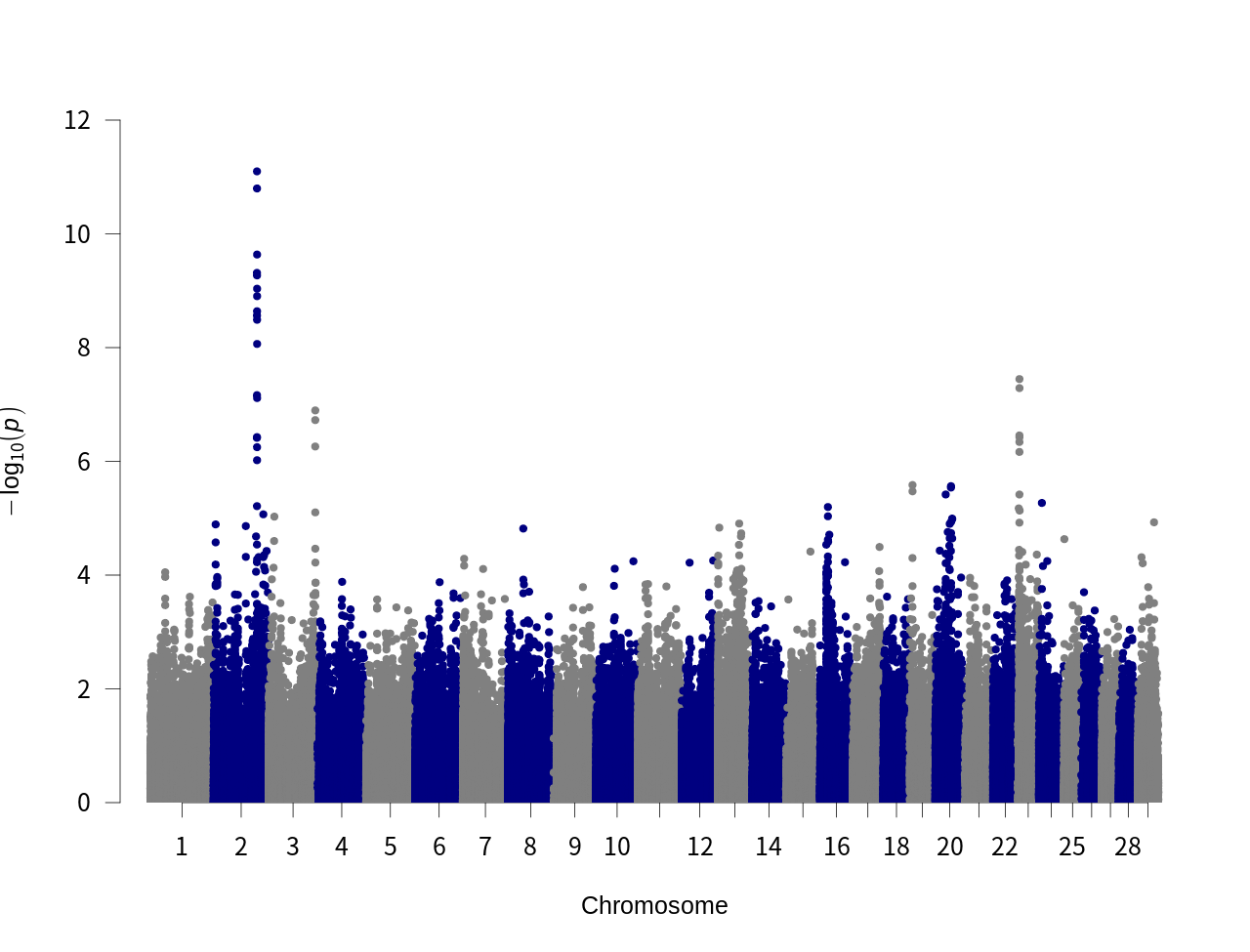

Supplement: Supplementary Figure 1 — Venn diagram of 1,000 (A), 3,000 (B), 5,000 (C), and 10,000 (D) pre-selected variants from WGS overlapping between traits. Backfat thickness (BFT), carcass weight (CWT), longissimus muscle area (LMA), and marbling score (MS). [file Data_Sheet_1.ZIP › Figure 2 - Manhattan_tif/Figure 2A - ITR_BFT.tiff]

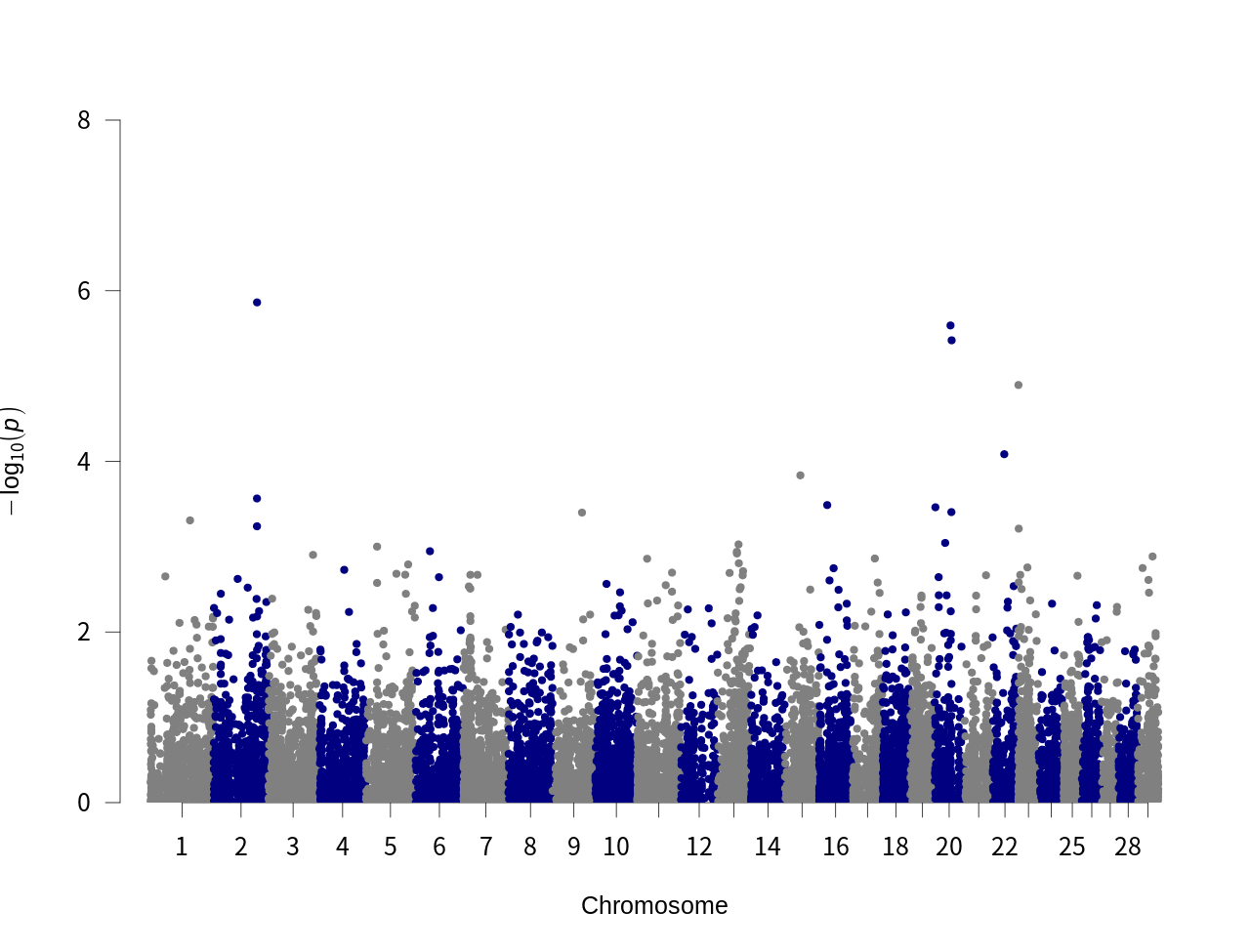

Supplement: Supplementary Figure 1 — Venn diagram of 1,000 (A), 3,000 (B), 5,000 (C), and 10,000 (D) pre-selected variants from WGS overlapping between traits. Backfat thickness (BFT), carcass weight (CWT), longissimus muscle area (LMA), and marbling score (MS). [file Data_Sheet_1.ZIP › Figure 2 - Manhattan_tif/Figure 2A - NSY_BFT.tiff]

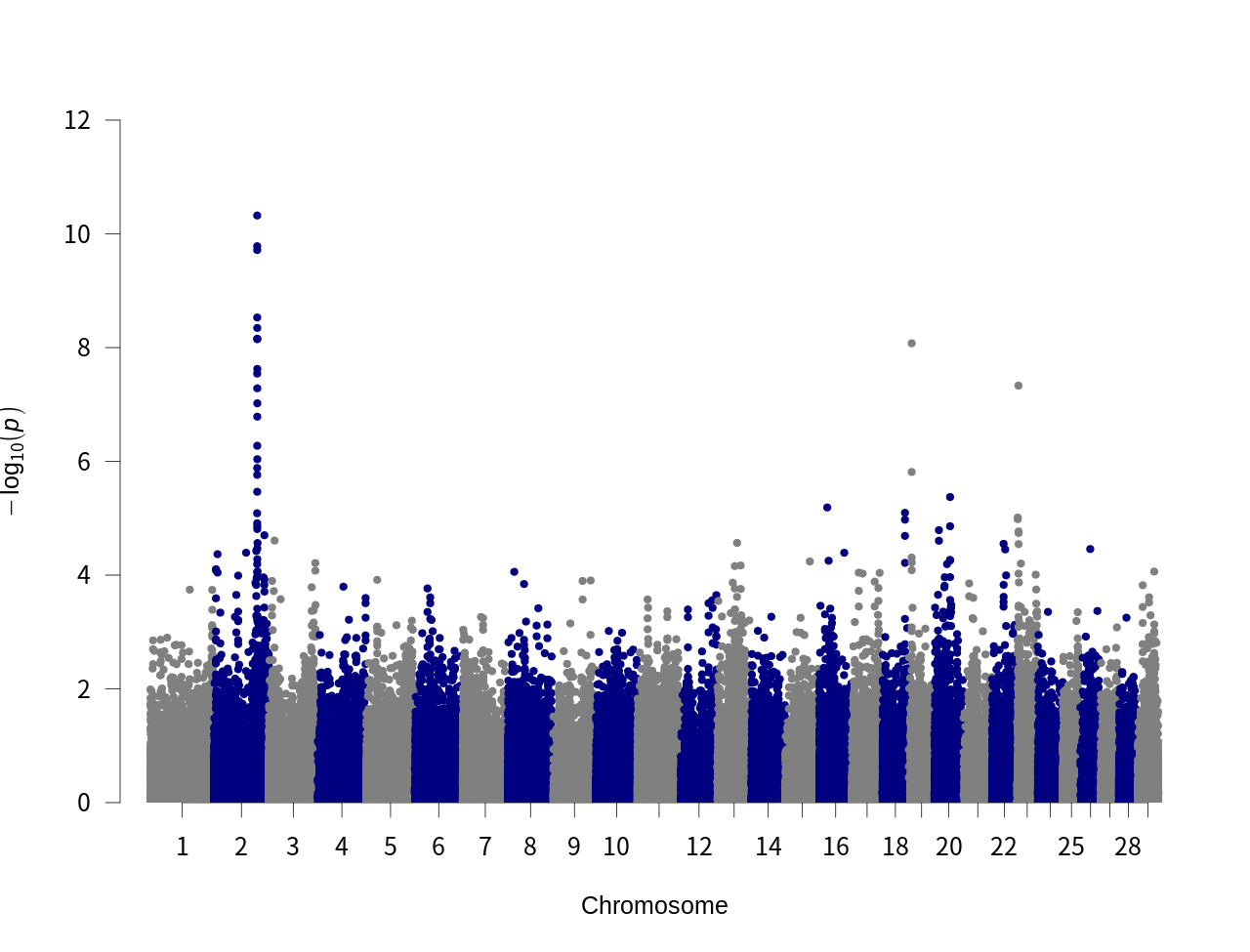

Supplement: Supplementary Figure 1 — Venn diagram of 1,000 (A), 3,000 (B), 5,000 (C), and 10,000 (D) pre-selected variants from WGS overlapping between traits. Backfat thickness (BFT), carcass weight (CWT), longissimus muscle area (LMA), and marbling score (MS). [file Data_Sheet_1.ZIP › Figure 2 - Manhattan_tif/Figure 2A - REG_BFT.tiff]

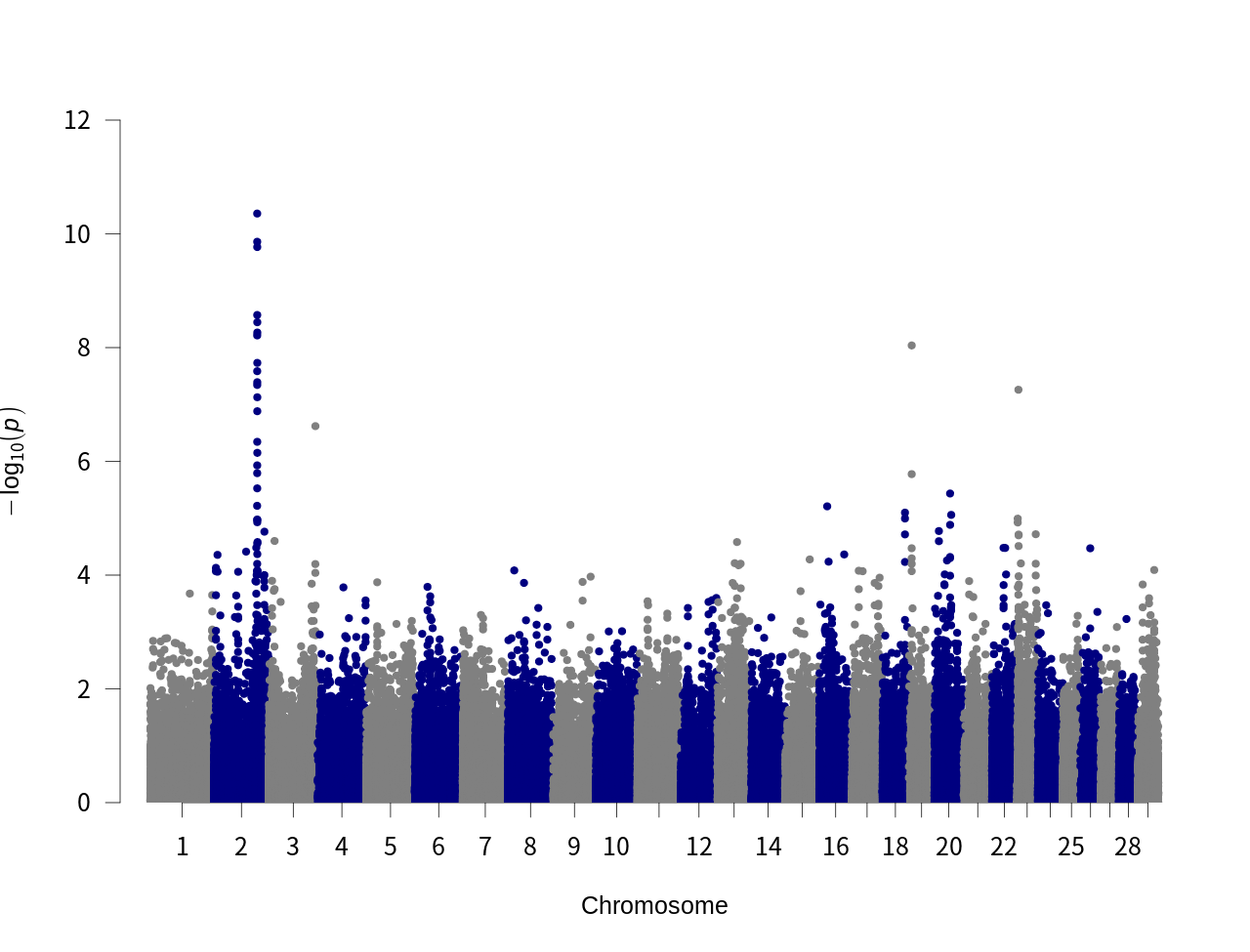

Supplement: Supplementary Figure 1 — Venn diagram of 1,000 (A), 3,000 (B), 5,000 (C), and 10,000 (D) pre-selected variants from WGS overlapping between traits. Backfat thickness (BFT), carcass weight (CWT), longissimus muscle area (LMA), and marbling score (MS). [file Data_Sheet_1.ZIP › Figure 2 - Manhattan_tif/Figure 2A - RSN_BFT.tiff]

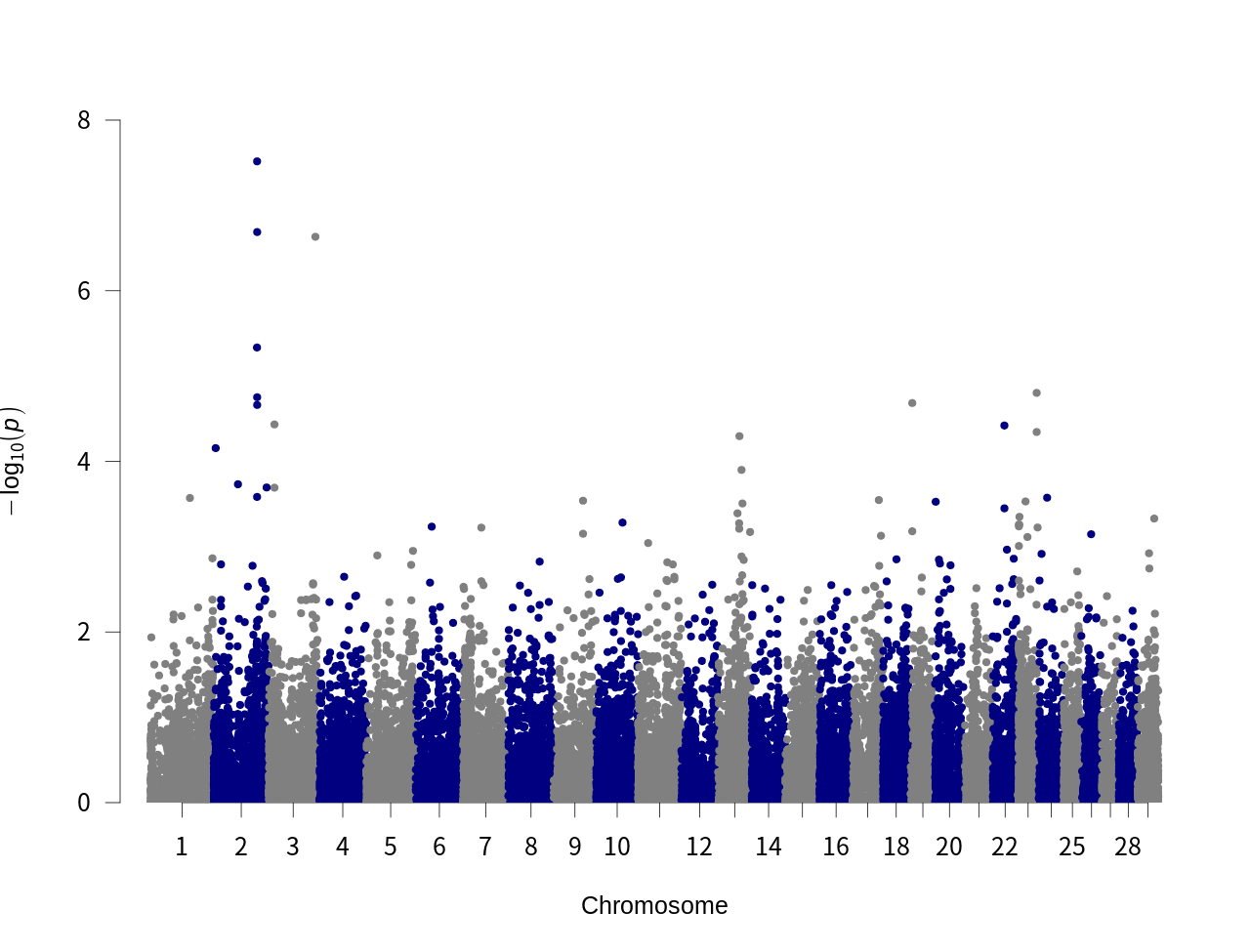

Supplement: Supplementary Figure 1 — Venn diagram of 1,000 (A), 3,000 (B), 5,000 (C), and 10,000 (D) pre-selected variants from WGS overlapping between traits. Backfat thickness (BFT), carcass weight (CWT), longissimus muscle area (LMA), and marbling score (MS). [file Data_Sheet_1.ZIP › Figure 2 - Manhattan_tif/Figure 2A - SYN_BFT.tiff]

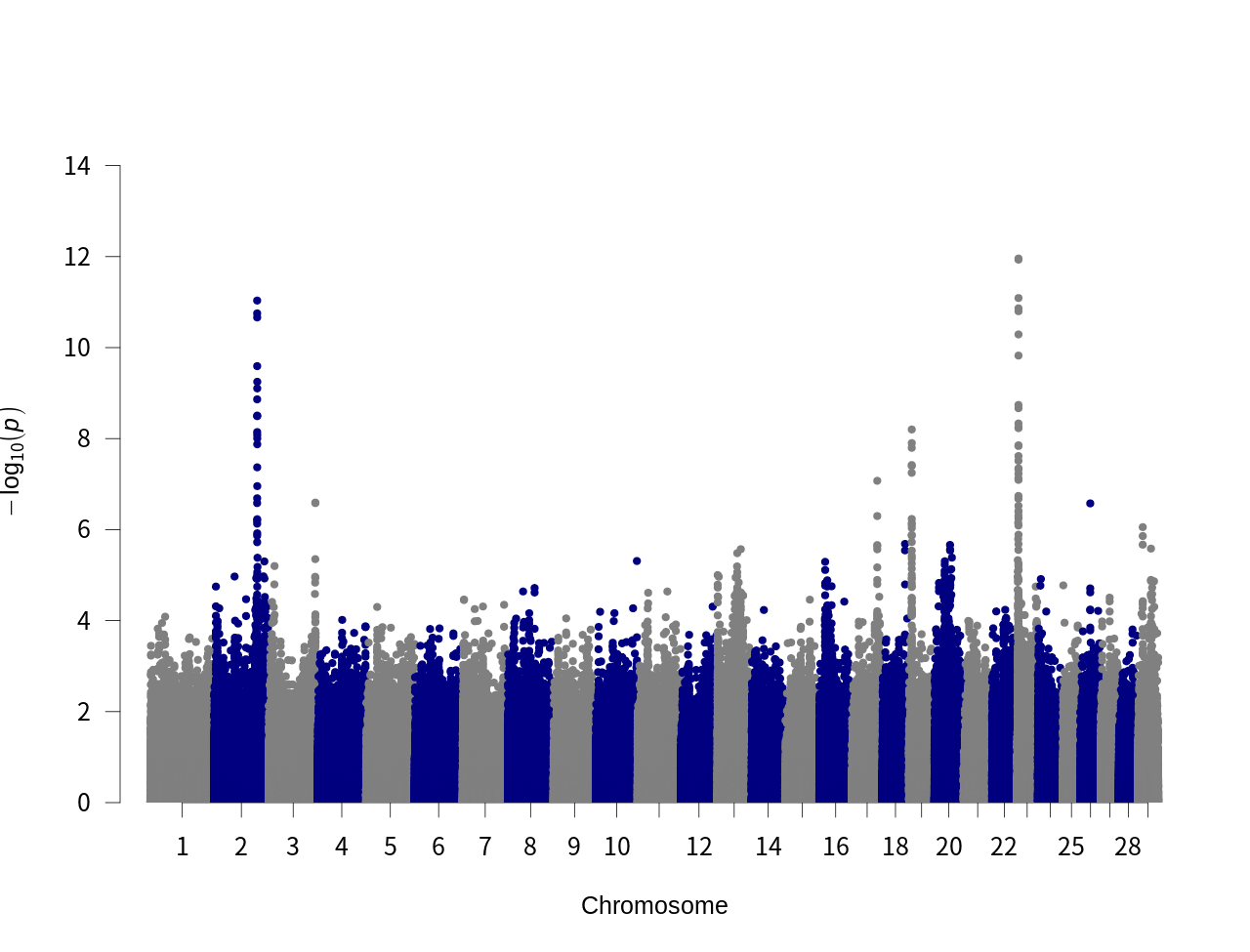

Supplement: Supplementary Figure 1 — Venn diagram of 1,000 (A), 3,000 (B), 5,000 (C), and 10,000 (D) pre-selected variants from WGS overlapping between traits. Backfat thickness (BFT), carcass weight (CWT), longissimus muscle area (LMA), and marbling score (MS). [file Data_Sheet_1.ZIP › Figure 2 - Manhattan_tif/Figure 2A - WGS_BFT.tiff]

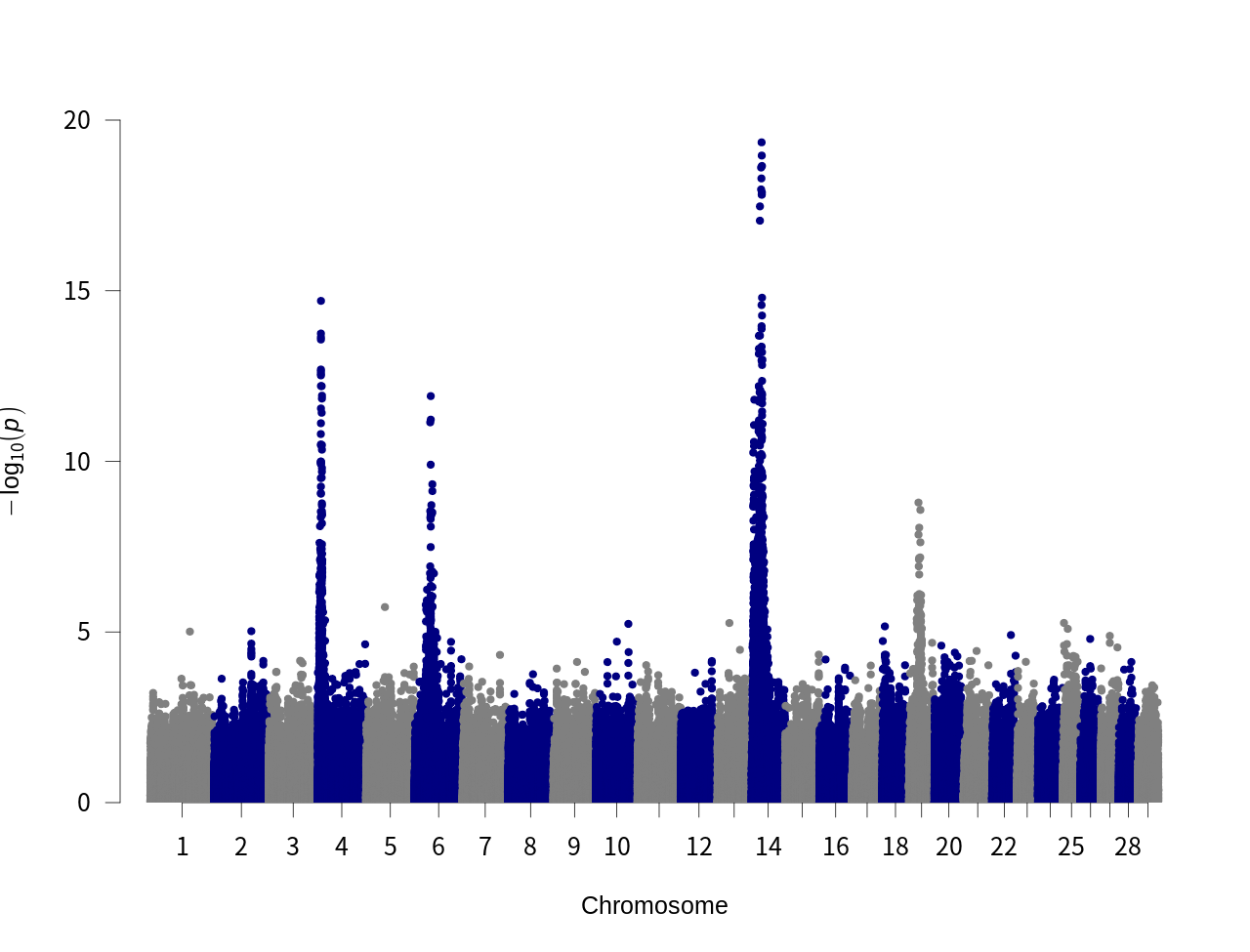

Supplement: Supplementary Figure 1 — Venn diagram of 1,000 (A), 3,000 (B), 5,000 (C), and 10,000 (D) pre-selected variants from WGS overlapping between traits. Backfat thickness (BFT), carcass weight (CWT), longissimus muscle area (LMA), and marbling score (MS). [file Data_Sheet_1.ZIP › Figure 2 - Manhattan_tif/Figure 2B - IGR_CWT.tiff]

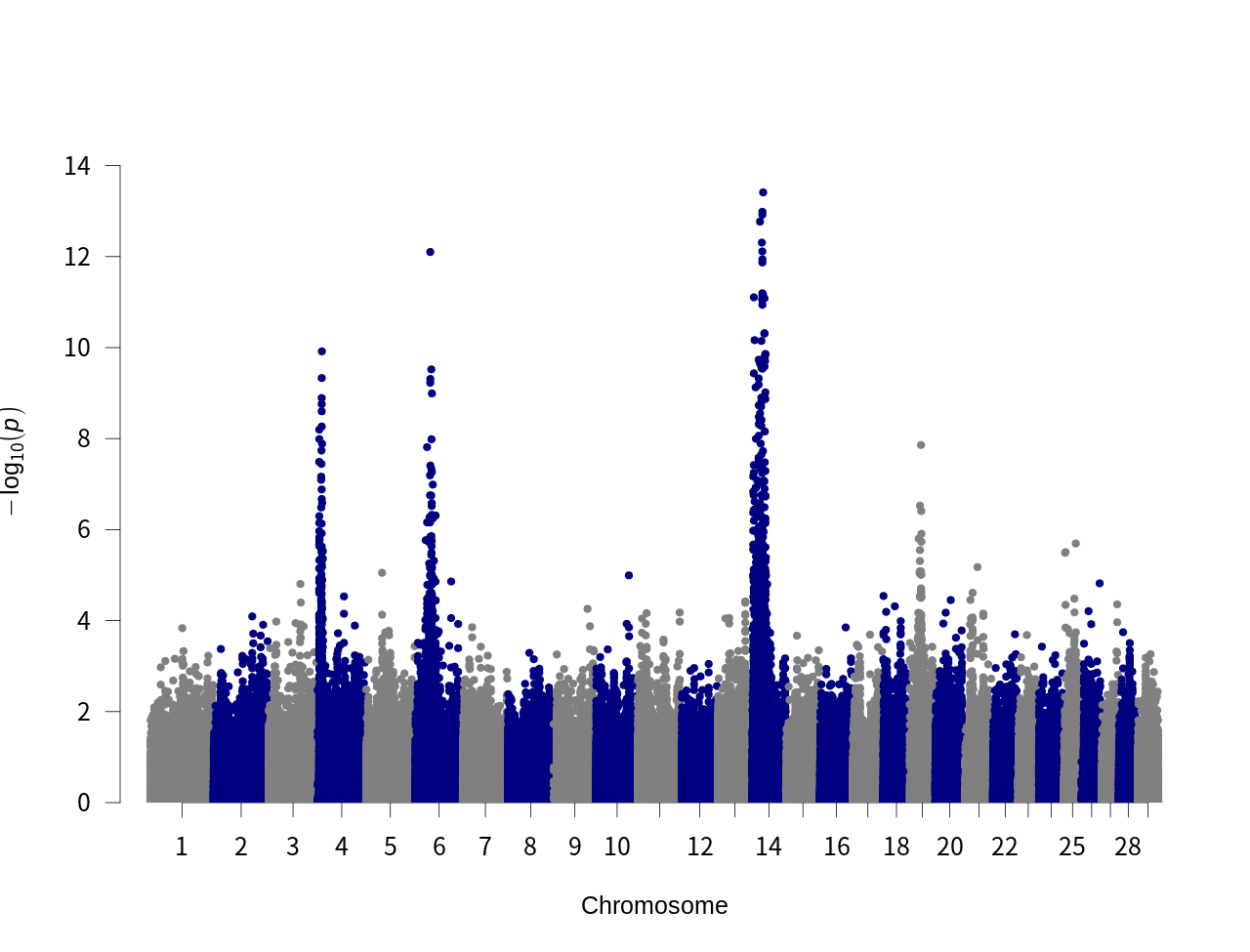

Supplement: Supplementary Figure 1 — Venn diagram of 1,000 (A), 3,000 (B), 5,000 (C), and 10,000 (D) pre-selected variants from WGS overlapping between traits. Backfat thickness (BFT), carcass weight (CWT), longissimus muscle area (LMA), and marbling score (MS). [file Data_Sheet_1.ZIP › Figure 2 - Manhattan_tif/Figure 2B - ITR_CWT.tiff]

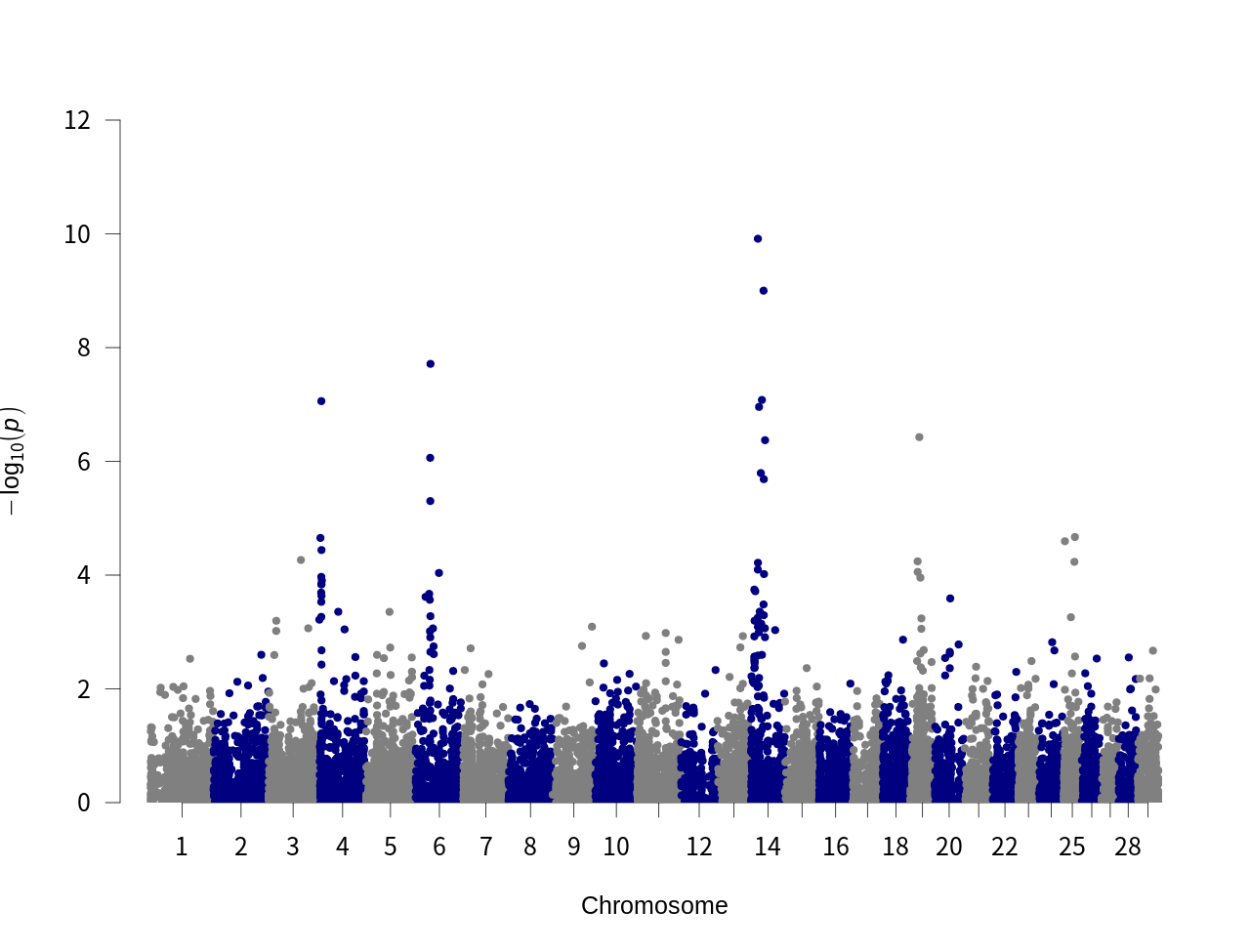

Supplement: Supplementary Figure 1 — Venn diagram of 1,000 (A), 3,000 (B), 5,000 (C), and 10,000 (D) pre-selected variants from WGS overlapping between traits. Backfat thickness (BFT), carcass weight (CWT), longissimus muscle area (LMA), and marbling score (MS). [file Data_Sheet_1.ZIP › Figure 2 - Manhattan_tif/Figure 2B - NSY_CWT.tiff]

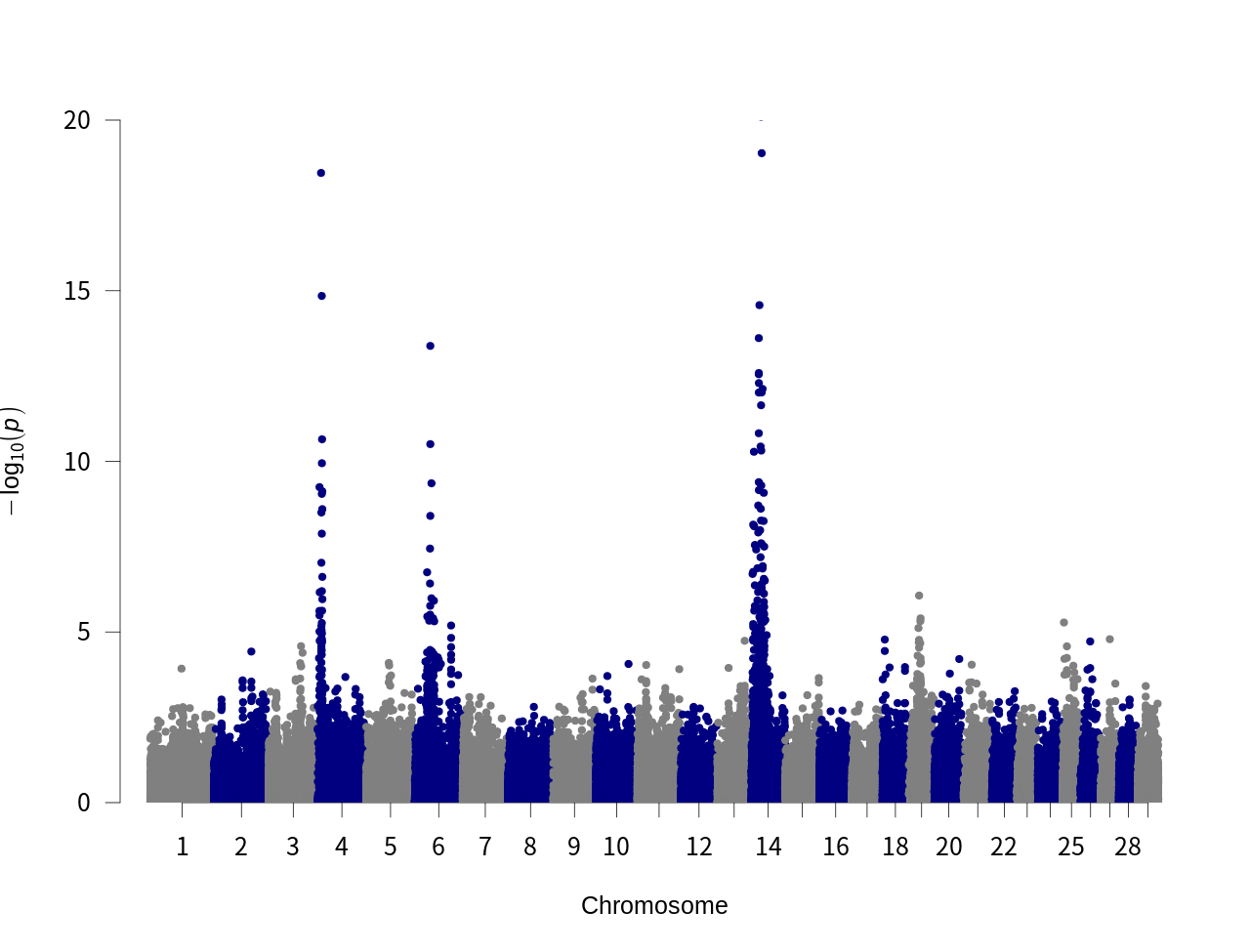

Supplement: Supplementary Figure 1 — Venn diagram of 1,000 (A), 3,000 (B), 5,000 (C), and 10,000 (D) pre-selected variants from WGS overlapping between traits. Backfat thickness (BFT), carcass weight (CWT), longissimus muscle area (LMA), and marbling score (MS). [file Data_Sheet_1.ZIP › Figure 2 - Manhattan_tif/Figure 2B - REG_CWT.tiff]

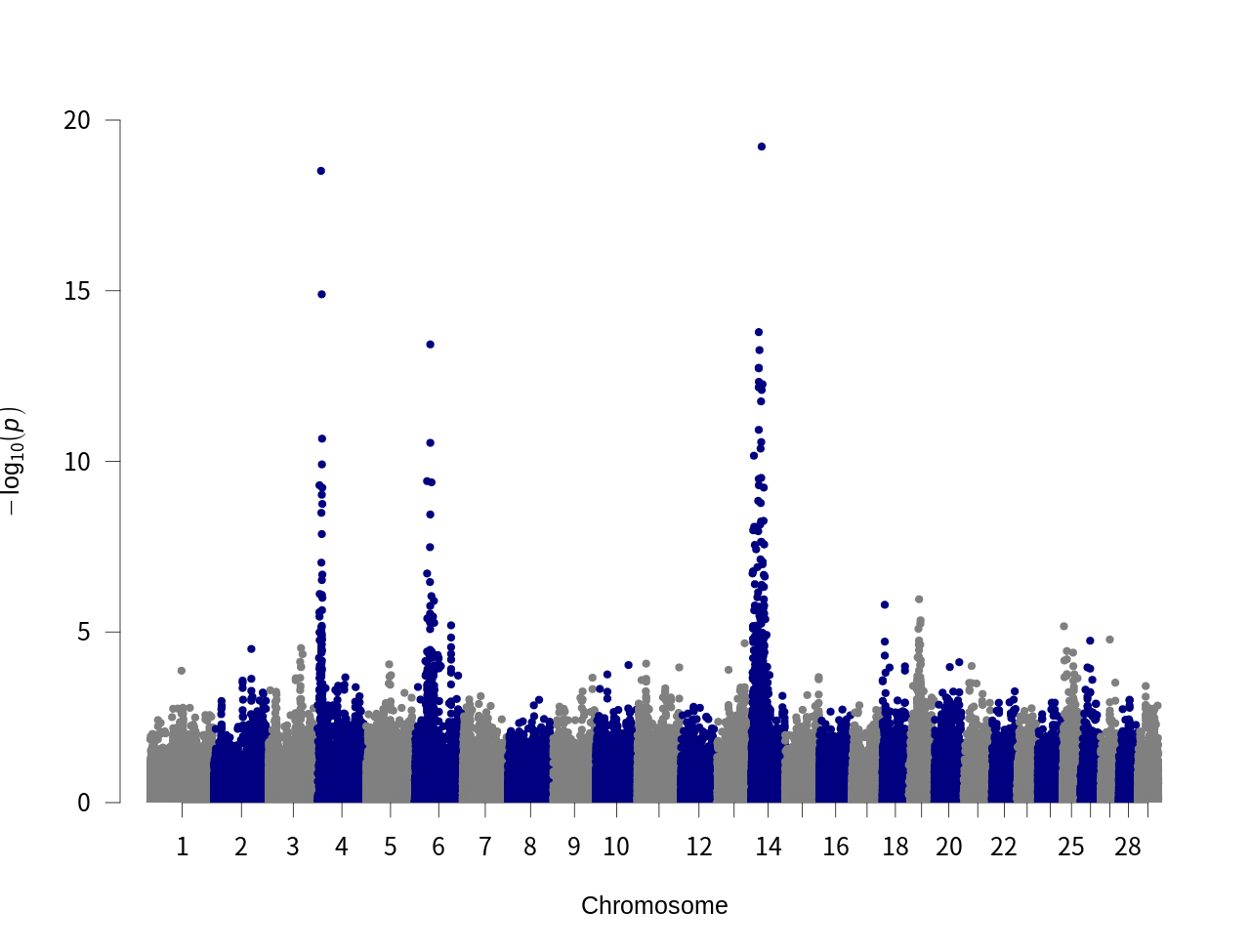

Supplement: Supplementary Figure 1 — Venn diagram of 1,000 (A), 3,000 (B), 5,000 (C), and 10,000 (D) pre-selected variants from WGS overlapping between traits. Backfat thickness (BFT), carcass weight (CWT), longissimus muscle area (LMA), and marbling score (MS). [file Data_Sheet_1.ZIP › Figure 2 - Manhattan_tif/Figure 2B - RSN_CWT.tiff]

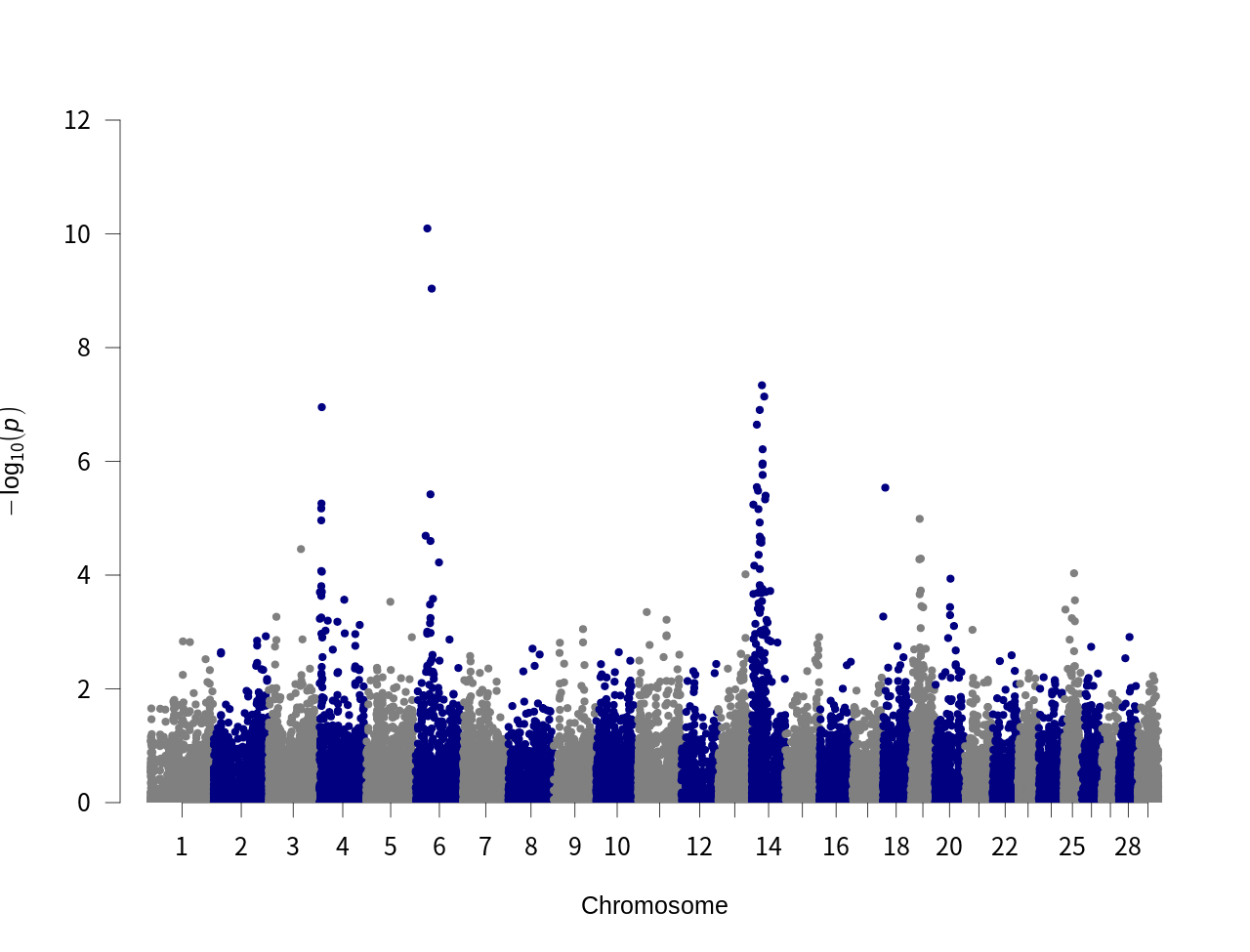

Supplement: Supplementary Figure 1 — Venn diagram of 1,000 (A), 3,000 (B), 5,000 (C), and 10,000 (D) pre-selected variants from WGS overlapping between traits. Backfat thickness (BFT), carcass weight (CWT), longissimus muscle area (LMA), and marbling score (MS). [file Data_Sheet_1.ZIP › Figure 2 - Manhattan_tif/Figure 2B - SYN_CWT.tiff]

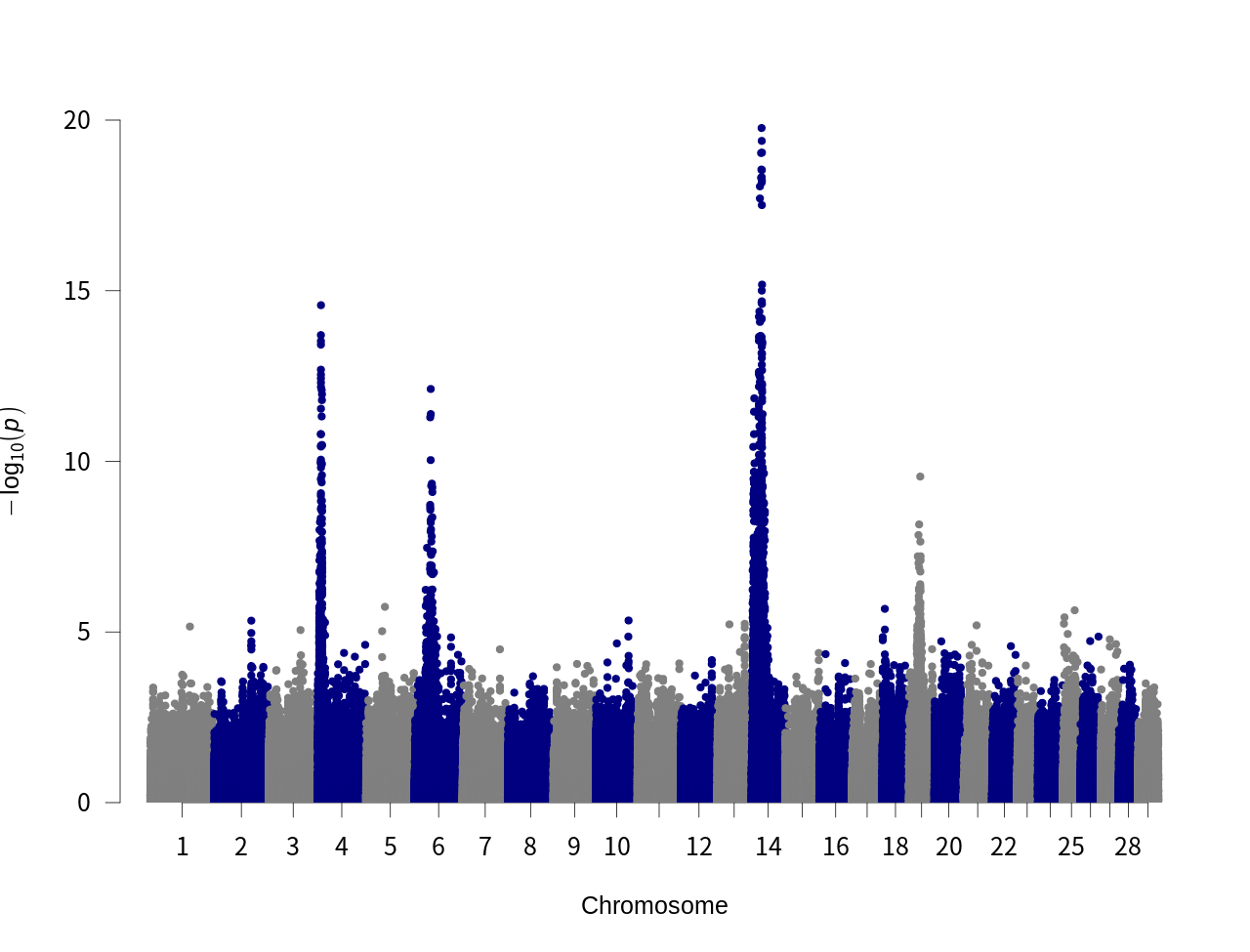

Supplement: Supplementary Figure 1 — Venn diagram of 1,000 (A), 3,000 (B), 5,000 (C), and 10,000 (D) pre-selected variants from WGS overlapping between traits. Backfat thickness (BFT), carcass weight (CWT), longissimus muscle area (LMA), and marbling score (MS). [file Data_Sheet_1.ZIP › Figure 2 - Manhattan_tif/Figure 2B - WGS_CWT.tiff]

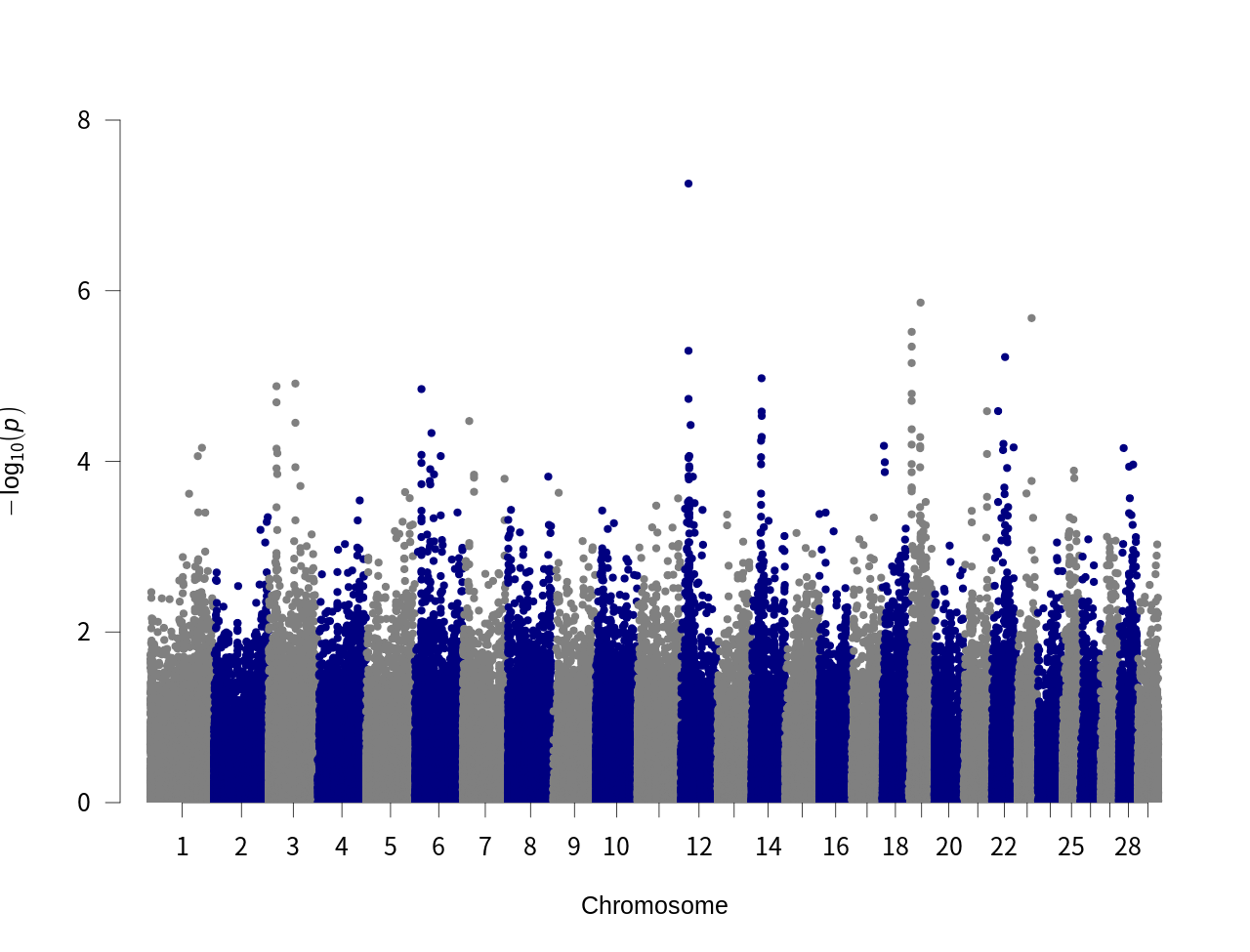

Supplement: Supplementary Figure 1 — Venn diagram of 1,000 (A), 3,000 (B), 5,000 (C), and 10,000 (D) pre-selected variants from WGS overlapping between traits. Backfat thickness (BFT), carcass weight (CWT), longissimus muscle area (LMA), and marbling score (MS). [file Data_Sheet_1.ZIP › Figure 2 - Manhattan_tif/Figure 2C - REG_EMA.tiff]

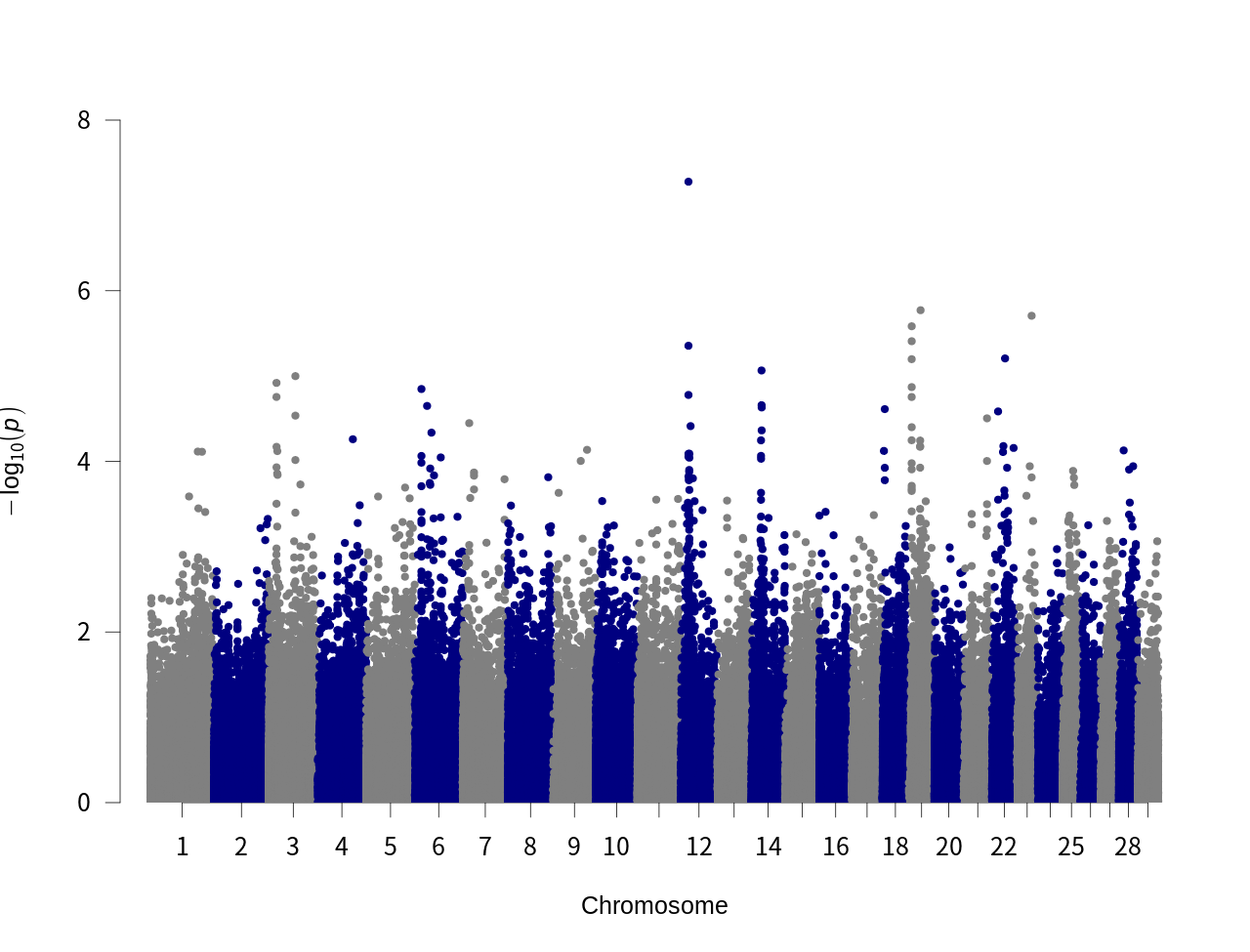

Supplement: Supplementary Figure 1 — Venn diagram of 1,000 (A), 3,000 (B), 5,000 (C), and 10,000 (D) pre-selected variants from WGS overlapping between traits. Backfat thickness (BFT), carcass weight (CWT), longissimus muscle area (LMA), and marbling score (MS). [file Data_Sheet_1.ZIP › Figure 2 - Manhattan_tif/Figure 2C - RSN_EMA.tiff]

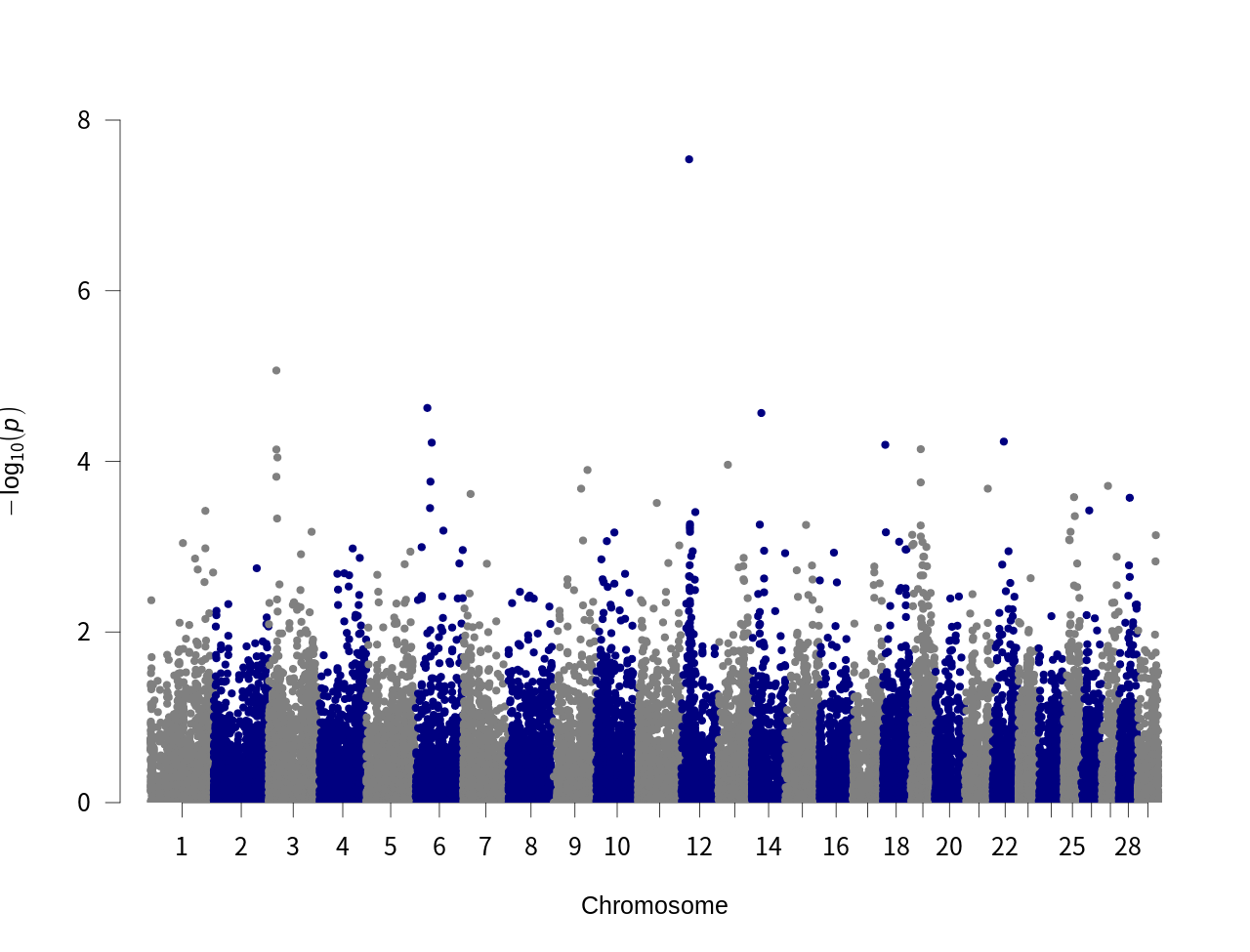

Supplement: Supplementary Figure 1 — Venn diagram of 1,000 (A), 3,000 (B), 5,000 (C), and 10,000 (D) pre-selected variants from WGS overlapping between traits. Backfat thickness (BFT), carcass weight (CWT), longissimus muscle area (LMA), and marbling score (MS). [file Data_Sheet_1.ZIP › Figure 2 - Manhattan_tif/Figure 2C - SYN_EMA.tiff]

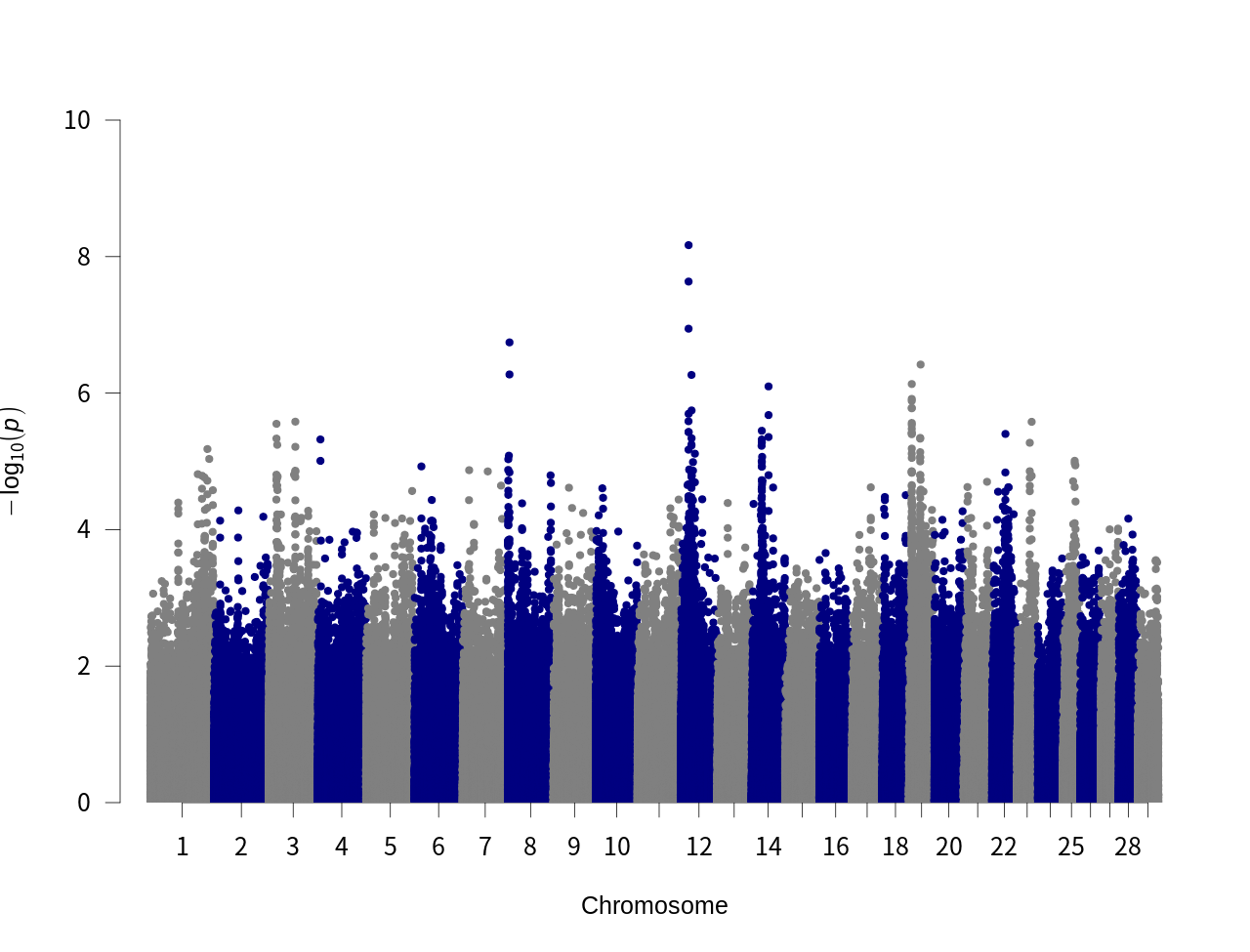

Supplement: Supplementary Figure 1 — Venn diagram of 1,000 (A), 3,000 (B), 5,000 (C), and 10,000 (D) pre-selected variants from WGS overlapping between traits. Backfat thickness (BFT), carcass weight (CWT), longissimus muscle area (LMA), and marbling score (MS). [file Data_Sheet_1.ZIP › Figure 2 - Manhattan_tif/Figure 2C - WGS_EMA.tiff]

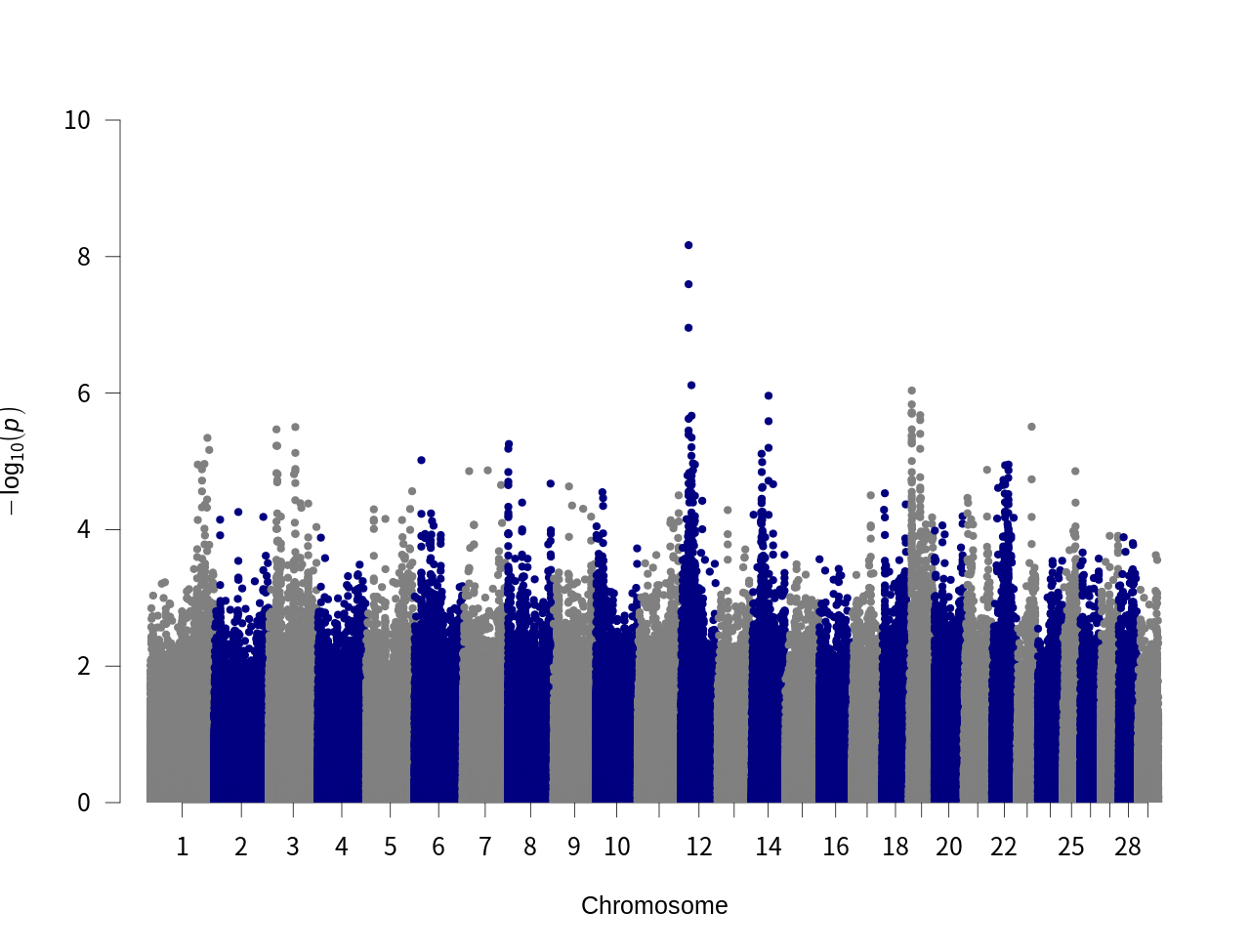

Supplement: Supplementary Figure 1 — Venn diagram of 1,000 (A), 3,000 (B), 5,000 (C), and 10,000 (D) pre-selected variants from WGS overlapping between traits. Backfat thickness (BFT), carcass weight (CWT), longissimus muscle area (LMA), and marbling score (MS). [file Data_Sheet_1.ZIP › Figure 2 - Manhattan_tif/Figure 2C - IGR_EMA.tiff]

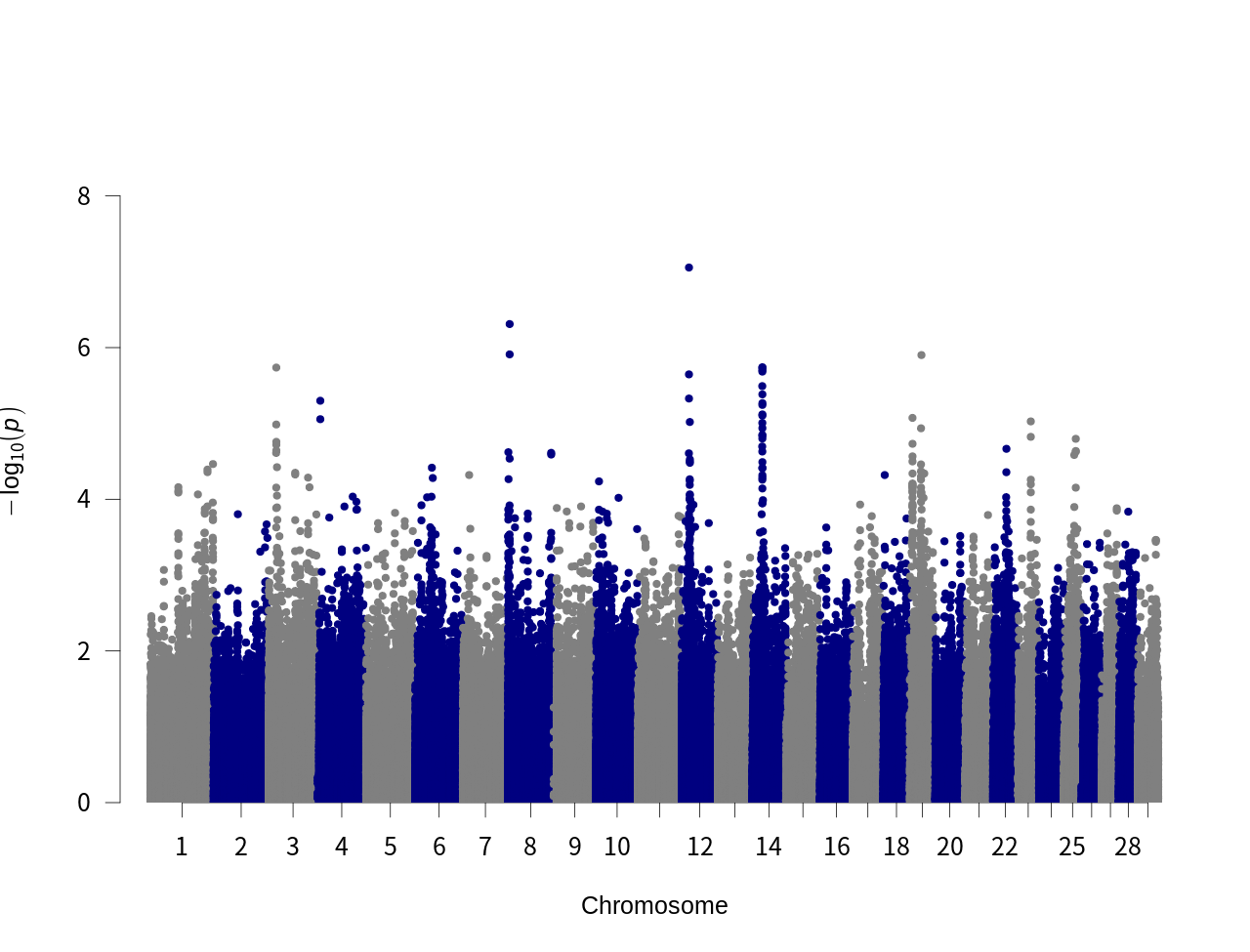

Supplement: Supplementary Figure 1 — Venn diagram of 1,000 (A), 3,000 (B), 5,000 (C), and 10,000 (D) pre-selected variants from WGS overlapping between traits. Backfat thickness (BFT), carcass weight (CWT), longissimus muscle area (LMA), and marbling score (MS). [file Data_Sheet_1.ZIP › Figure 2 - Manhattan_tif/Figure 2C - ITR_EMA.tiff]

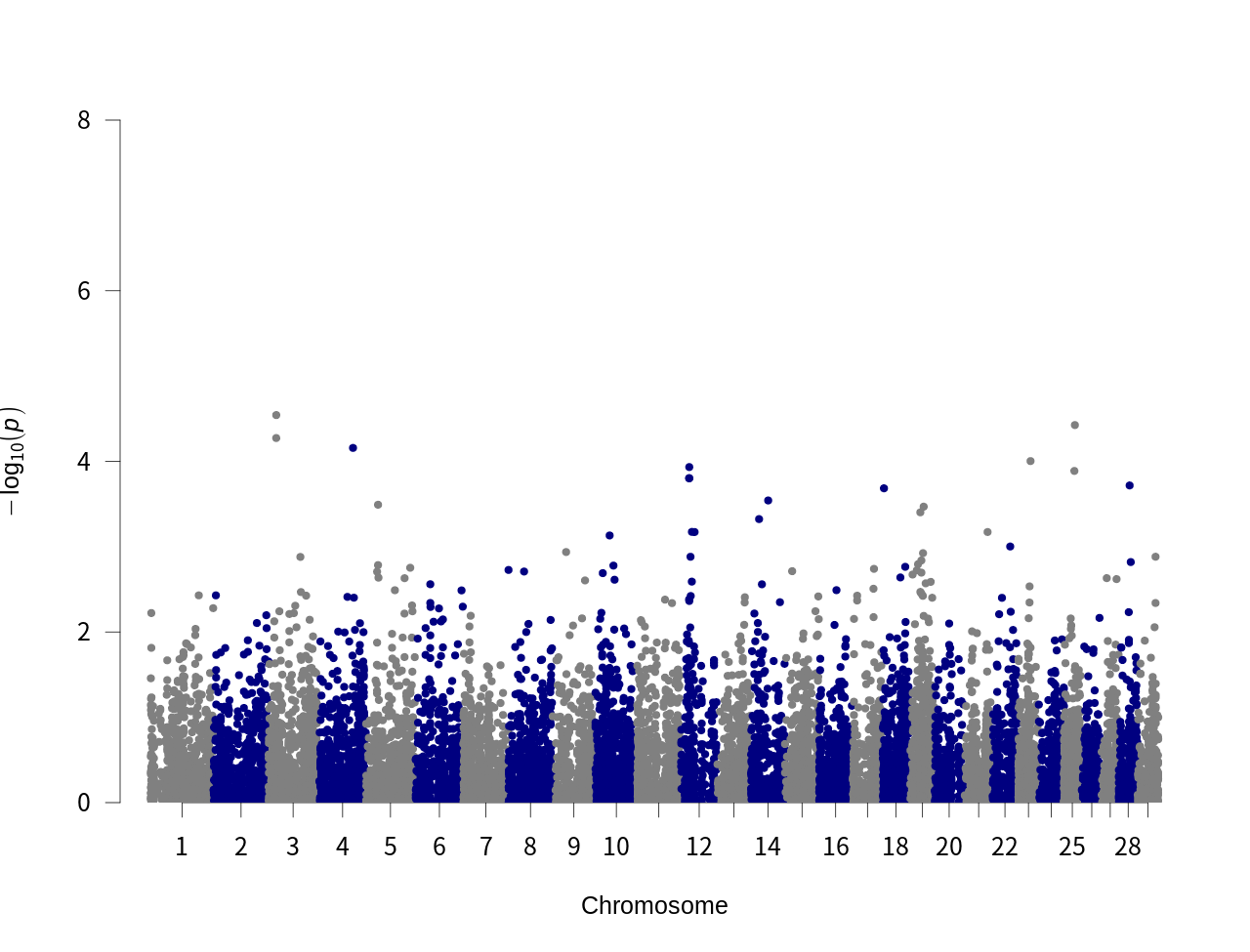

Supplement: Supplementary Figure 1 — Venn diagram of 1,000 (A), 3,000 (B), 5,000 (C), and 10,000 (D) pre-selected variants from WGS overlapping between traits. Backfat thickness (BFT), carcass weight (CWT), longissimus muscle area (LMA), and marbling score (MS). [file Data_Sheet_1.ZIP › Figure 2 - Manhattan_tif/Figure 2C - NSY_EMA.tiff]

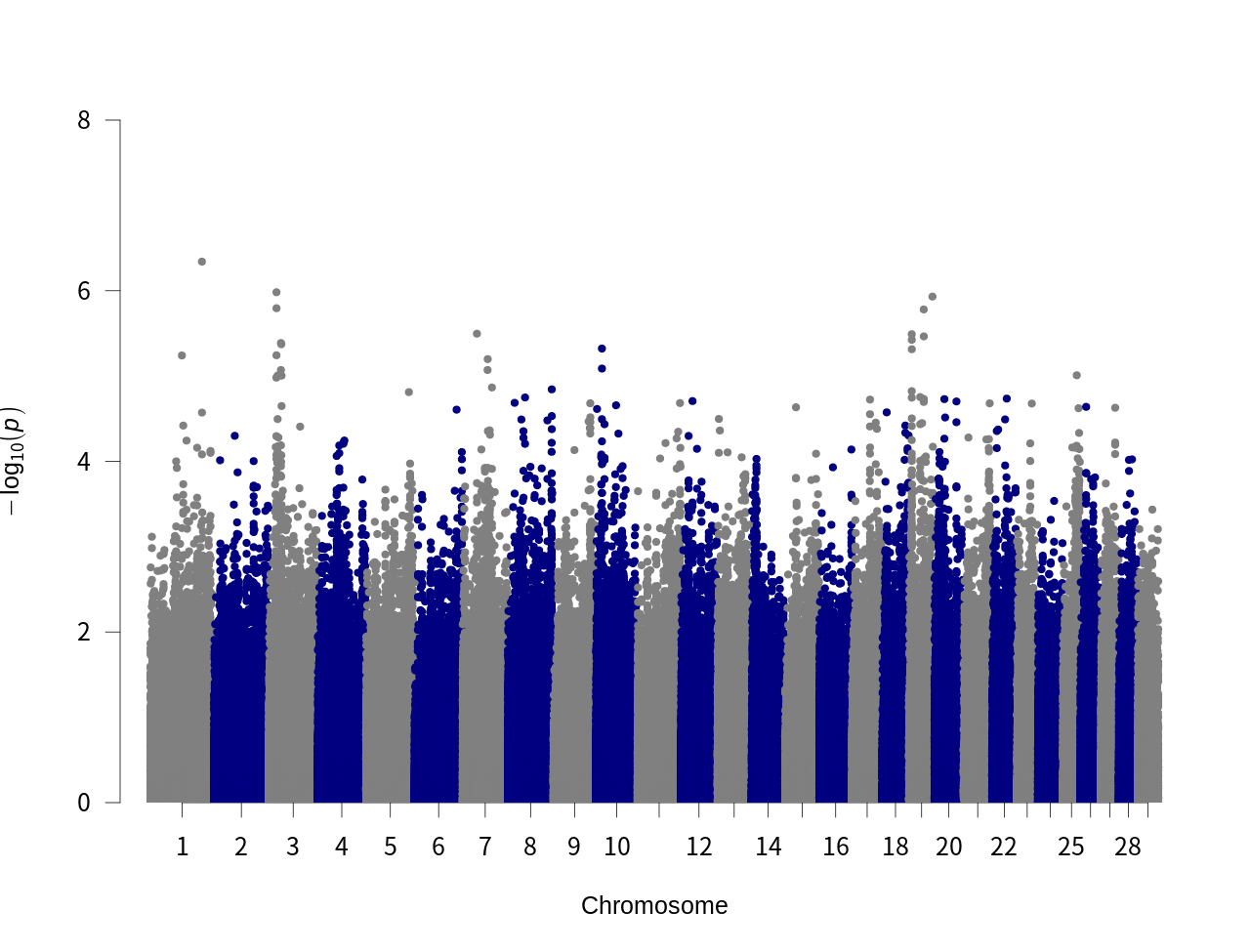

Supplement: Supplementary Figure 1 — Venn diagram of 1,000 (A), 3,000 (B), 5,000 (C), and 10,000 (D) pre-selected variants from WGS overlapping between traits. Backfat thickness (BFT), carcass weight (CWT), longissimus muscle area (LMA), and marbling score (MS). [file Data_Sheet_1.ZIP › Figure 2 - Manhattan_tif/Figure 2D - IGR_MS.tiff]

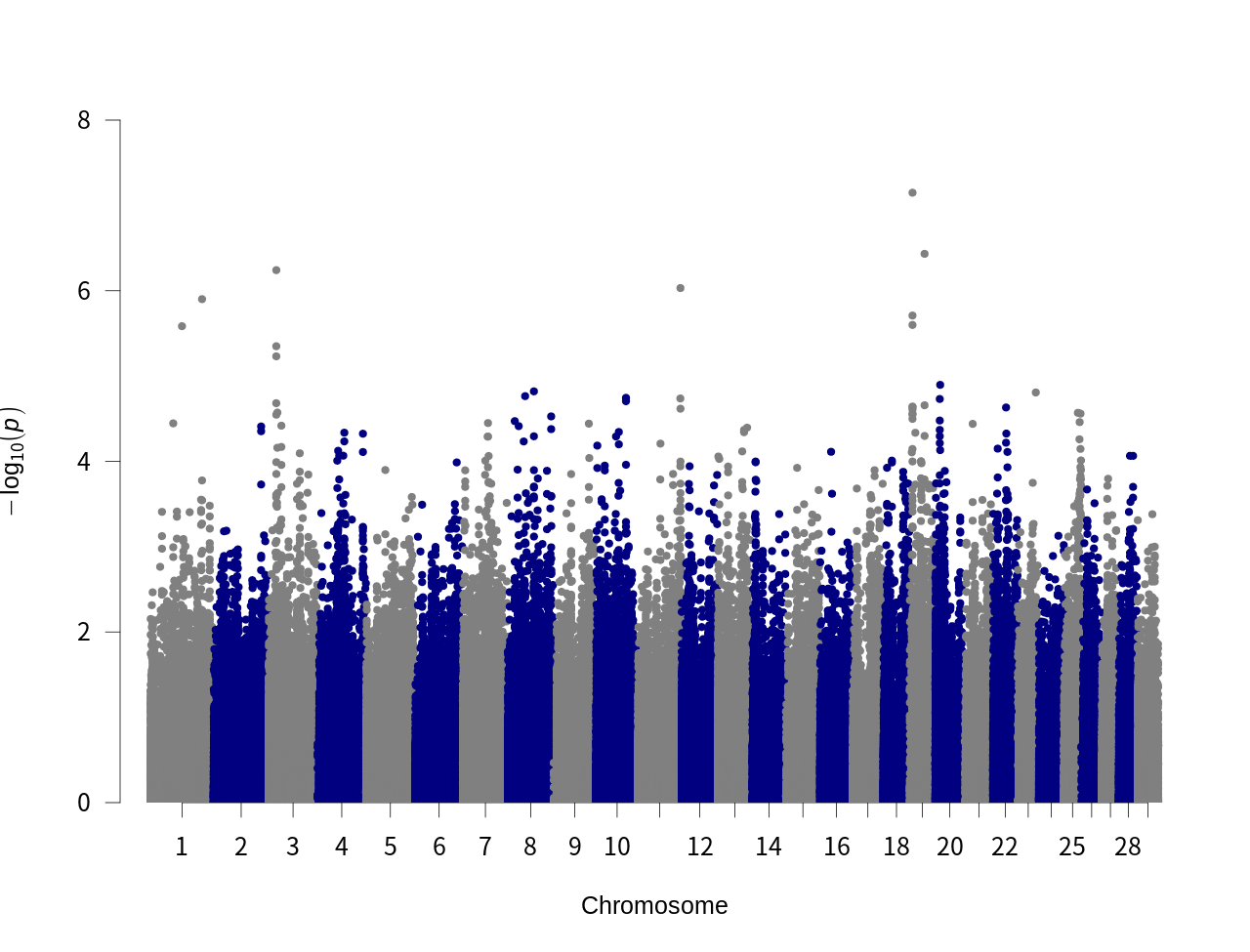

Supplement: Supplementary Figure 1 — Venn diagram of 1,000 (A), 3,000 (B), 5,000 (C), and 10,000 (D) pre-selected variants from WGS overlapping between traits. Backfat thickness (BFT), carcass weight (CWT), longissimus muscle area (LMA), and marbling score (MS). [file Data_Sheet_1.ZIP › Figure 2 - Manhattan_tif/Figure 2D - ITR_MS.tiff]

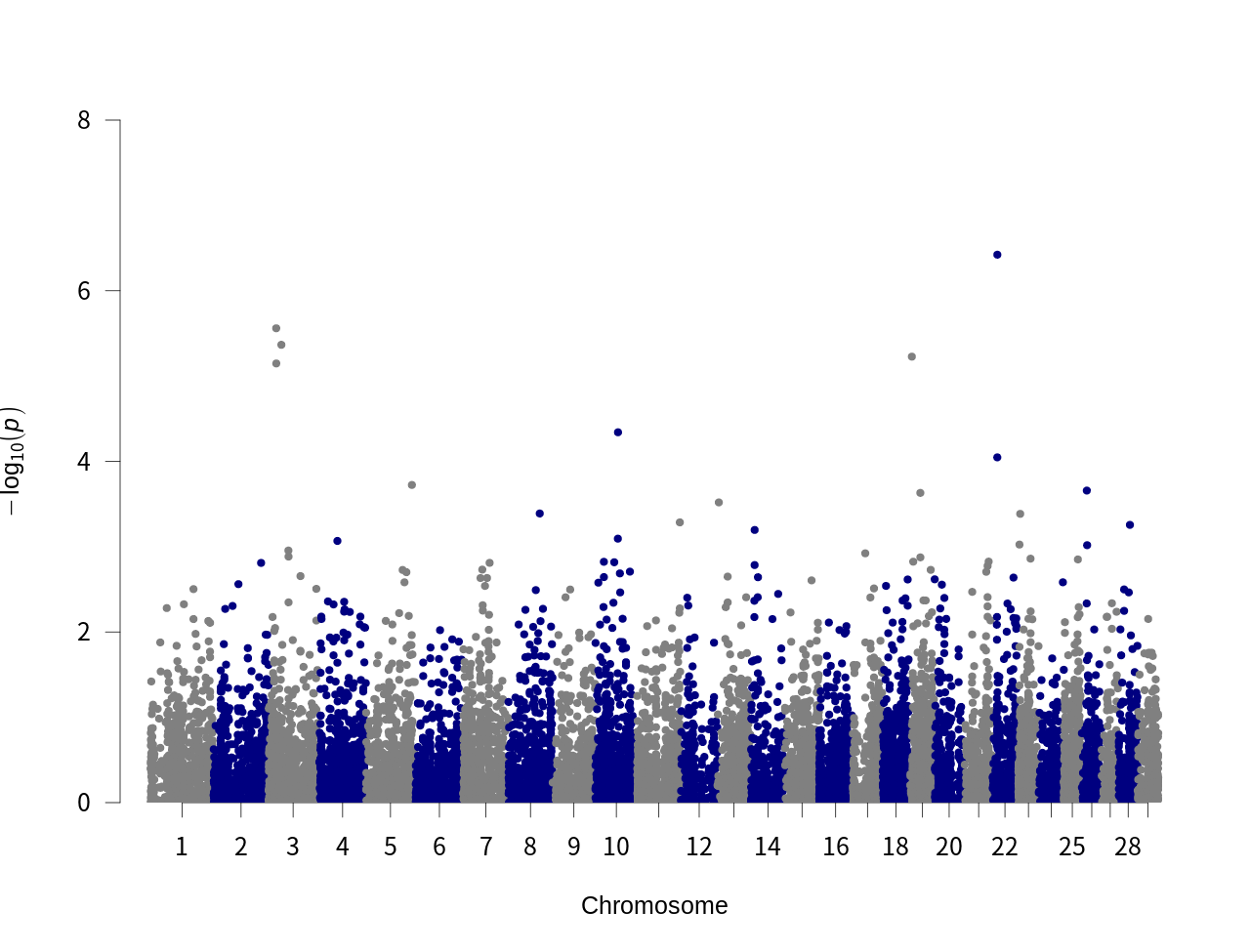

Supplement: Supplementary Figure 1 — Venn diagram of 1,000 (A), 3,000 (B), 5,000 (C), and 10,000 (D) pre-selected variants from WGS overlapping between traits. Backfat thickness (BFT), carcass weight (CWT), longissimus muscle area (LMA), and marbling score (MS). [file Data_Sheet_1.ZIP › Figure 2 - Manhattan_tif/Figure 2D - NSY_MS.tiff]

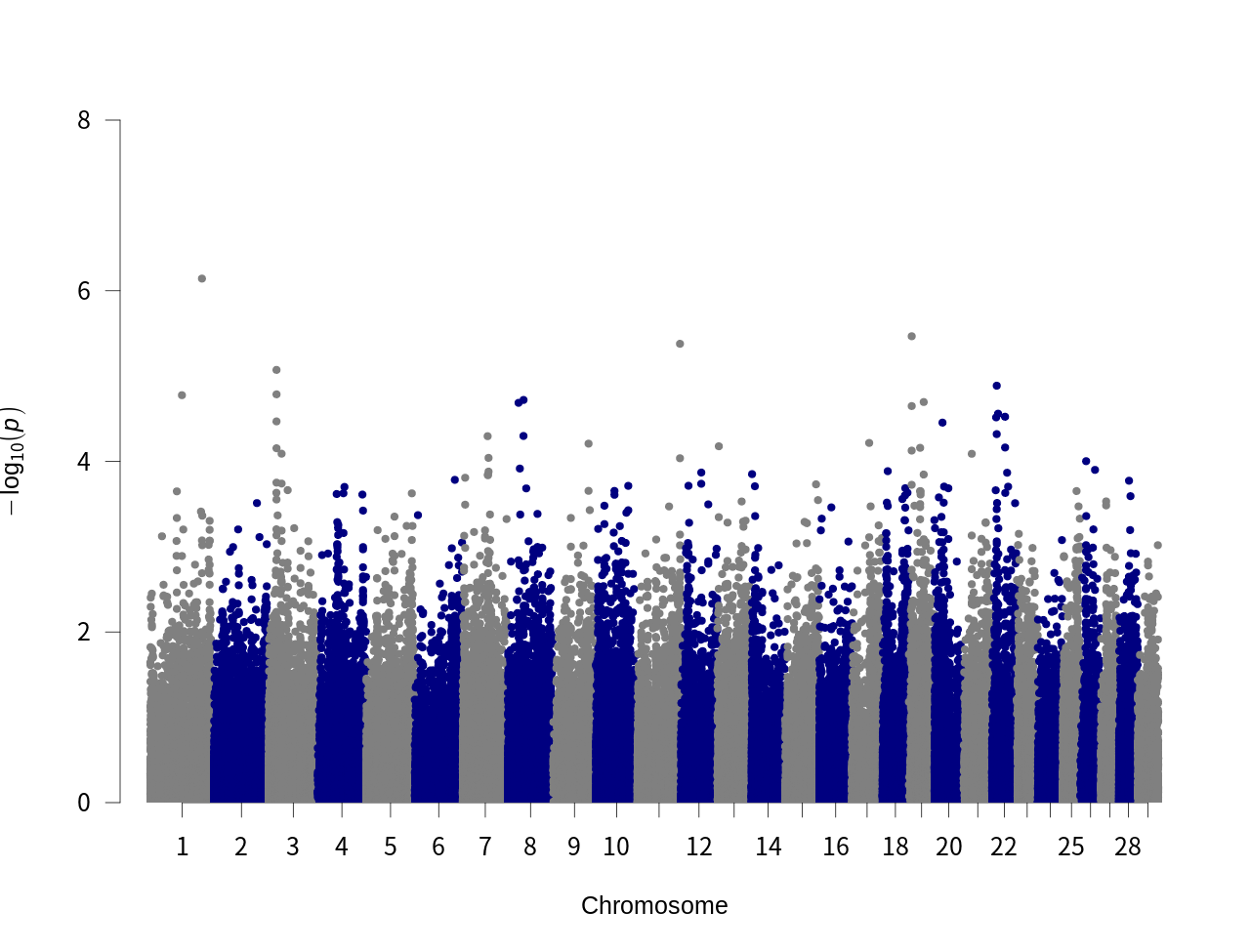

Supplement: Supplementary Figure 1 — Venn diagram of 1,000 (A), 3,000 (B), 5,000 (C), and 10,000 (D) pre-selected variants from WGS overlapping between traits. Backfat thickness (BFT), carcass weight (CWT), longissimus muscle area (LMA), and marbling score (MS). [file Data_Sheet_1.ZIP › Figure 2 - Manhattan_tif/Figure 2D - REG_MS.tiff]

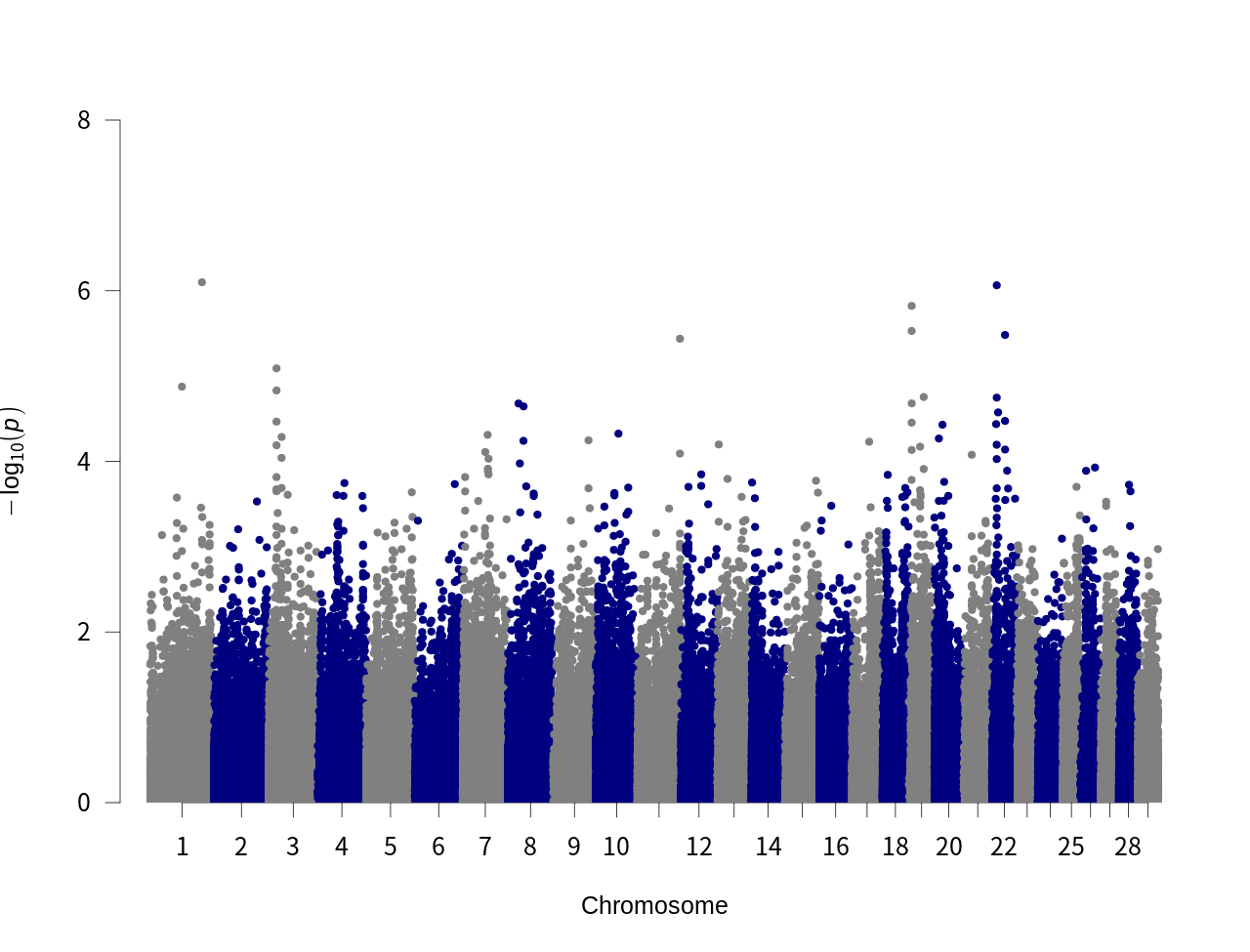

Supplement: Supplementary Figure 1 — Venn diagram of 1,000 (A), 3,000 (B), 5,000 (C), and 10,000 (D) pre-selected variants from WGS overlapping between traits. Backfat thickness (BFT), carcass weight (CWT), longissimus muscle area (LMA), and marbling score (MS). [file Data_Sheet_1.ZIP › Figure 2 - Manhattan_tif/Figure 2D - RSN_MS.tiff]

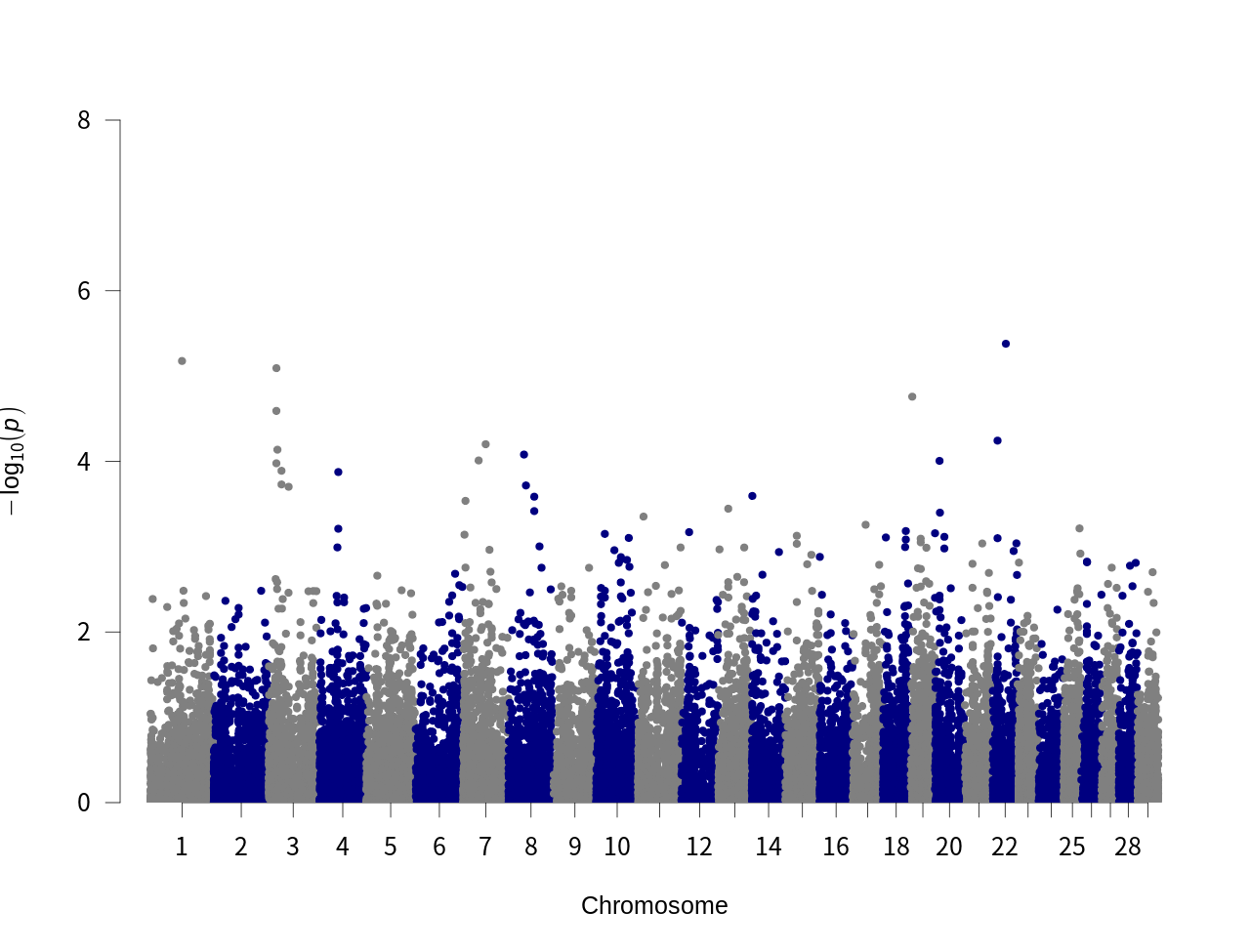

Supplement: Supplementary Figure 1 — Venn diagram of 1,000 (A), 3,000 (B), 5,000 (C), and 10,000 (D) pre-selected variants from WGS overlapping between traits. Backfat thickness (BFT), carcass weight (CWT), longissimus muscle area (LMA), and marbling score (MS). [file Data_Sheet_1.ZIP › Figure 2 - Manhattan_tif/Figure 2D - SYN_MS.tiff]

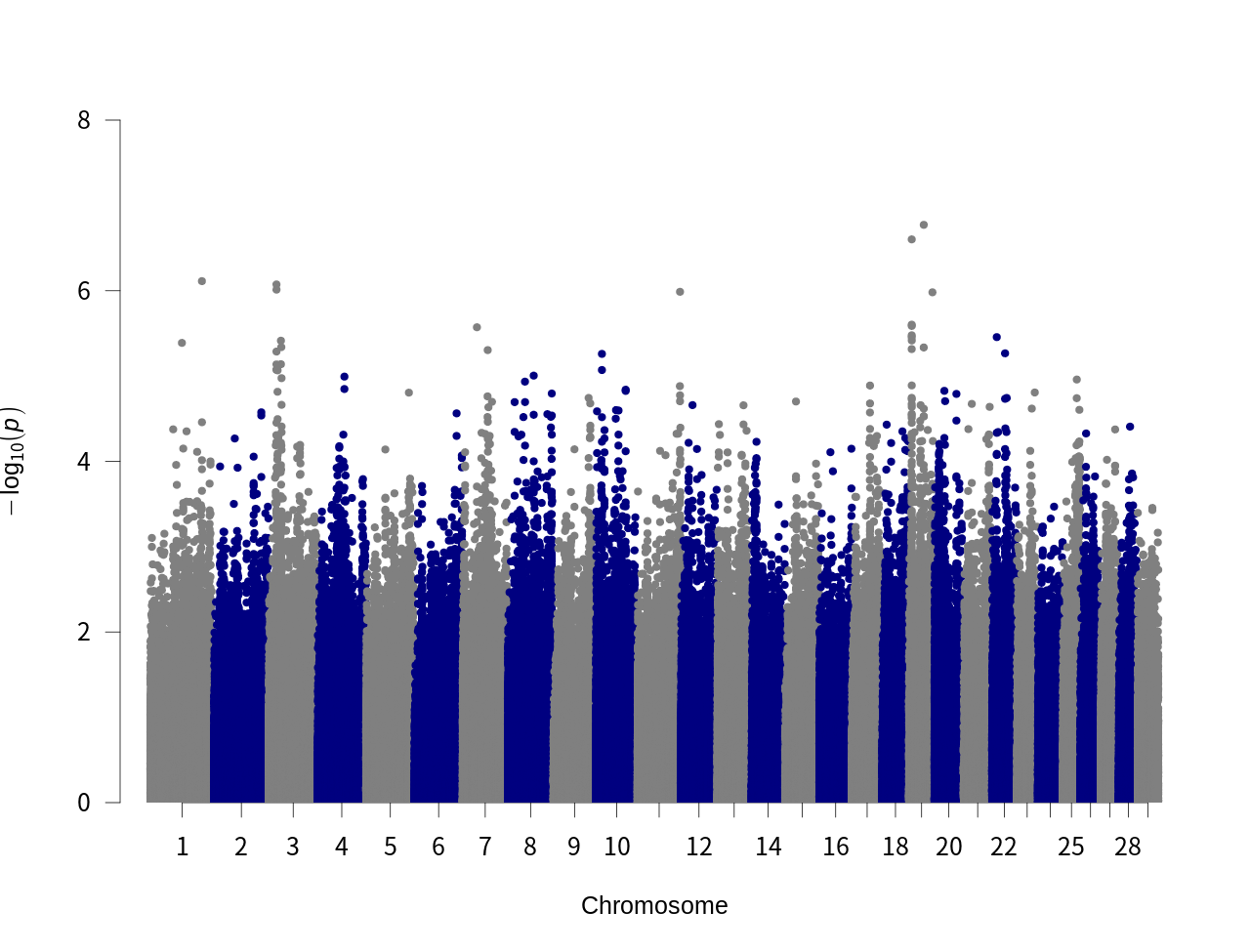

Supplement: Supplementary Figure 1 — Venn diagram of 1,000 (A), 3,000 (B), 5,000 (C), and 10,000 (D) pre-selected variants from WGS overlapping between traits. Backfat thickness (BFT), carcass weight (CWT), longissimus muscle area (LMA), and marbling score (MS). [file Data_Sheet_1.ZIP › Figure 2 - Manhattan_tif/Figure 2D - WGS_MS.tiff]
