# Supplementary material for: Genomic Prediction Based on SNP Functional Annotation Using Imputed Whole-Genome Sequence Data in Korean Hanwoo Cattle
Source: Front Genet. 2021 Jan 21;11:603822. doi: 10.3389/fgene.2020.603822 (PMC7859490; doi:10.3389/fgene.2020.603822)
Supplement: Supplementary Table 1 — Number of singleton animals based on different GRM relatedness threshold. [file Table_1.DOCX]

| GRM threshold | No. of Animals | |
| --- | --- | --- |
|  | Singleton | Family |
| 0.025 | 4 | 16,888 |
| 0.050 | 74 | 16,818 |
| 0.125 | 730 | 16,162 |
| 0.250 | 1,913 | 14,979 |
| 0.300 | 7,324 | 9,568 |
| 0.400 | 16,523 | 369 |
